# HIV-1 competition experiments in humanized mice show that APOBEC3H imposes selective pressure and promotes virus adaptation

**DOI:** 10.1371/journal.ppat.1006348

**Published:** 2017-05-05

**Authors:** Yusuke Nakano, Naoko Misawa, Guillermo Juarez-Fernandez, Miyu Moriwaki, Shinji Nakaoka, Takaaki Funo, Eri Yamada, Andrew Soper, Rokusuke Yoshikawa, Diako Ebrahimi, Yuuya Tachiki, Shingo Iwami, Reuben S. Harris, Yoshio Koyanagi, Kei Sato

**Affiliations:** 1 Laboratory of Systems Virology, Department of Biosystems Science, Institute for Frontier Life and Medical Sciences, Kyoto University, Kyoto, Japan; 2 Graduate School of Biostudies, Kyoto University, Kyoto, Japan; 3 Institute of Industrial Sciences, The University of Tokyo, Meguro-ku, Tokyo, Japan; 4 PRESTO, Japan Science and Technology Agency, Kawaguchi, Saitama, Japan; 5 Mathematical Biology Laboratory, Department of Biology, Faculty of Sciences, Kyushu University, Fukuoka, Japan; 6 Department of Biochemistry, Molecular Biology and Biophysics, University of Minnesota, Minneapolis, Minnesota, United States of America; 7 Masonic Cancer Center, University of Minnesota, Minneapolis, Minnesota, United States of America; 8 Institute for Molecular Virology, University of Minnesota, Minneapolis, Minnesota, United States of America; 9 Center for Genome Engineering, University of Minnesota, Minneapolis, Minnesota, United States of America; 10 CREST, Japan Science and Technology Agency, Kawaguchi, Saitama, Japan; 11 Howard Hughes Medical Institute, University of Minnesota, Minneapolis, Minnesota, United States of America; Duke University Medical Center, UNITED STATES

## Abstract

APOBEC3 (A3) family proteins are DNA cytosine deaminases recognized for contributing to HIV-1 restriction and mutation. Prior studies have demonstrated that A3D, A3F, and A3G enzymes elicit a robust anti-HIV-1 effect in cell cultures and in humanized mouse models. Human A3H is polymorphic and can be categorized into three phenotypes: stable, intermediate, and unstable. However, the anti-viral effect of endogenous A3H *in vivo* has yet to be examined. Here we utilize a hematopoietic stem cell-transplanted humanized mouse model and demonstrate that stable A3H robustly affects HIV-1 fitness *in vivo*. In contrast, the selection pressure mediated by intermediate A3H is relaxed. Intriguingly, viral genomic RNA sequencing reveled that HIV-1 frequently adapts to better counteract stable A3H during replication in humanized mice. Molecular phylogenetic analyses and mathematical modeling suggest that stable A3H may be a critical factor in human-to-human viral transmission. Taken together, this study provides evidence that stable variants of A3H impose selective pressure on HIV-1.

## Author summary

Human APOBEC3 family proteins are known as intrinsic defenses against HIV-1, whereas HIV-1 Vif counteracts APOBEC3-mediated anti-viral action. Using a hematopoietic stem cell-transplanted humanized mouse model, we demonstrated that endogenous APOBEC3D, APOBEC3F and APOBEC3G play pivotal roles in restricting HIV-1 replication *in vivo*. In addition to these three APOBEC3 family proteins, certain haplotypes of APOBEC3H have the ability to control HIV-1 replication in cell culture studies. However, the anti-viral effect of APOBEC3H polymorphism *in vivo* and in human population is yet to be addressed. Here we use a humanized mouse model to show that acquiring resistance to anti-viral APOBEC3H is necessary for HIV-1 replication. Together with phylogenetic analyses and mathematical modeling, we conclude that APOBEC3H is a critical determinant of HIV-1 replication within infected individuals and we propose that it may also be a factor in human-to-human HIV-1 transmission.

## Introduction

Apolipoprotein B mRNA editing enzyme catalytic polypeptide-like 3 (APOBEC3; A3) enzymes are cellular single-stranded DNA cytosine deaminases that are specifically encoded in mammals [1,2]. Rodents including mice (*Mus musculus*) have a single *A3* gene, while primates including humans (*Homo sapiens*), chimpanzees (*Pan troglodytes*) and Old World monkeys have seven *A3* paralogous genes (*A3A*, *A3B*, *A3C*, *A3D*, *A3F*, *A3G* and *A3H*). Gene duplication is a hallmark of the genes that are under evolutionary selective pressures [3], and indeed, the seven primate *A3* genes have been positively selected during evolution [4], These observations suggest that primate A3 proteins play crucial roles in primates including humans. Human A3G was discovered first and was shown to be capable of restricting the replication of human immunodeficiency virus type 1 (HIV-1) in an *in vitro* cell culture system [5]. Subsequent investigations revealed that several human A3 family proteins exhibit the ability to reduce HIV-1 infectivity [2,6–8]. Moreover, previous studies including ours have demonstrated that A3D, A3F, and A3G, which are endogenously expressed in human CD4^+^ T cells, are restriction factors potently controlling HIV-1 replication in human CD34^+^ hematopoietic stem cell (HSC)-transplanted humanized mouse models [9–12]. To antagonize the anti-viral effect of A3 proteins, HIV-1 encodes a protein named viral infectivity factor (Vif). Vif orchestrates cellular ubiquitin ligase complex and degrades anti-viral A3 proteins via ubiquitin/proteasome-dependent pathway in infected cells [2,13].

**Fig 1:**
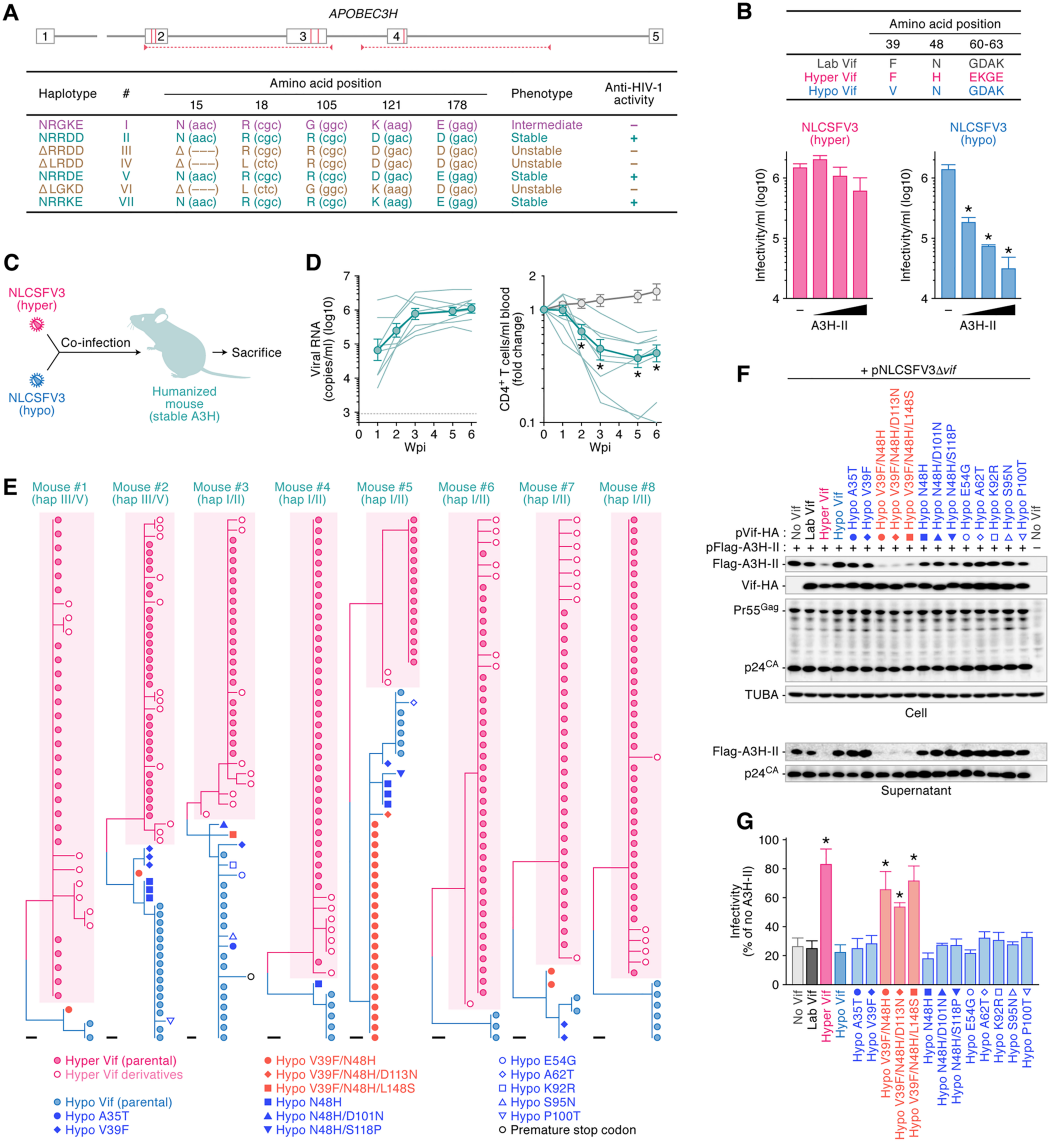
Dynamics of hyper/hypo HIV-1 infection in stable A3H humanized mice. (**A**) A schematic of the *A3H* gene locus and the 5 polymorphisms in *A3H* exons 2, 3, and 4 (indicated by red vertical lines) that combine to produce 7 different haplotypes. Red arrowheads with horizontal broken lines indicate the region amplified by genotyping PCR. In the bottom panel, the 7 different A3H haplotypes based on observed protein stability or instability in previous studies [14,15,20] are summarized. (**B**) (top) A schematic of the Vif protein encoded by HIV-1 used in this study. This panel shows the amino acid differences responsible for the degradation of stable A3H. (bottom) TZM-bl assay. The infectivity of released virions was determined by using TZM-bl cells. *P < 0.05 versus no A3H-II by Student's *t* test. The assay was performed in triplicate. The data represents average with SD. (**C, D**) HIV-1 infection in humanized mice. (**C**) A schematic of co-inoculation of hyper and hypo HIV-1s into stable A3H humanized mice. (**D**) Hyper and hypo viruses containing 2.5 ng of p24 antigen each (5 ng in total; n = 8) or RPMI1640 (n = 12; for mock infection) were inoculated into humanized mice. the amount of viral RNA in plasma (left) and the level of peripheral CD4^+^ T cells (CD45^+^ CD3^+^ CD4^+^ cells) (right) were analyzed at 0, 1, 2, 3, 5, and 6 wpi as described in Materials and Methods. The averages are shown in circles with SEMs, and the values from each mouse are shown by line. X-axes, wpi. In the left panel, horizontal broken line indicates detection limit (800 copies/ml plasma). In the right panel, *P < 0.05 versus mock-infected mice by Mann-Whitney U test. (**E**) Phylogenetic trees of *vif* sequence. Viral *vif* sequences in the plasma of infected mice at 6 wpi were analyzed as described in Materials and Methods. Results of each infected mouse (mice #1–8) are respectively shown. Each symbol represents identical sequence. Pink shadow indicates hyper *vif* derivatives. Scale bar represents one nucleotide substitution. Note that the 3 hypo *vif-*related sequences with the ability to counteract A3H-II (shown in **Fig 1F & 1G**) are indicated with red symbols. (**F, G**) Evaluation of anti-stable A3H activity of Vif derivatives detected in infected humanized mice. (**F**) Western blotting. The input of cell lysate was standardized to α-Tubulin (TUBA), and representative results are shown. (**G**) TZM-bl assay. The expression plasmids of the Vif derivatives were cotransfected with pNLCSFV3Δ*vif* and either with or without Flag-tagged A3H-II expression plasmid into HEK293T cells. The infectivity of released virus was determined by using TZM-bl cells, and the percentage of the value of "no A3H-II" is shown. *P < 0.05 versus "no Vif" by Student's *t* test. The assay was performed in triplicate. The data represents average with SD. In panels **F** and **G**, the symbols are identical to those in **Fig 1E**.

In addition to A3D, A3F and A3G, human A3H is known as a potent restriction factor against HIV-1. Human A3H is polymorphic and has seven haplotypes [14,15]. Three of them, called haplotypes II, V, and VII, produce stably expressed enzymes that exhibit anti-HIV-1 activity in model cell culture experiments as well as primary T lymphocytes *ex vivo* [14–16]. In contrast, the other three haplotypes (III, IV, and VI) do not exhibit detectable protein expression [14–16]. Additionally, our recent study has demonstrated that A3H haplotype I (A3H-I) has intermediate stability and clear enzymatic activity [17] (Fig 1A). Importantly, the frequency of each haplotype differs among human population, with a higher frequency of stable A3H in the African-descendant population [14,15]. Furthermore, it is more intriguing that the Vif proteins of certain HIV-1 strains are unable to counteract stable A3H haplotypes, and the ability of Vif to antagonize stable A3H is determined by at least two residues at positions 39 and 48 (Fig 1B) [18–20]. These observations suggest that both the A3H-mediated anti-viral effect and the antagonistic ability of Vif against A3H are co-mingled in the human population, in contrast to the functional relationships between Vif and A3D, A3F and A3G, which appear much less variable. However, the robustness of the effects of stable/intermediate A3H haplotypes on viral replication at an individual scale and a population scale remains unclear, and the dynamics by which HIV-1 may circumvent and/or counteract the anti-viral effect of stable A3H is yet to be addressed.

Here we use an HSC-transplanted humanized mouse model to demonstrate that stable A3H, but not intermediate A3H, which is endogenously expressed in human CD4^+^ T cells, is a *bona fide* restriction factor capable of controlling HIV-1 replication *in vivo*. In addition, we reveal that HIV-1 Vif readily acquires the ability to counteract stable A3H during viral expansion *in vivo*. Additionally, we use molecular phylogenetic analysis and mathematical modeling to further address the impact of stable A3H on HIV-1 epidemics. Our analyses suggest that stable A3H may control HIV-1 dissemination in both intra- and inter-individual scales.

## Results

### Endogenous A3H exhibits robust anti-viral effect *in vivo*

To address the impact of endogenous A3H haplotypes (Fig 1A) on HIV-1 replication *in vivo*, two derivatives of the replication-competent CCR5-tropic HIV-1 strain NLCSFV3 [21] were made with differing A3H haplotype II (A3H-II) neutralization capabilities [20]. One virus encodes a Vif protein that is able to counteract stable A3H ("hyper Vif"), while the other encodes a Vif protein that does not ("hypo Vif") (Fig 1B) [20]. Importantly, previous reports demonstrated that the Vif's ability to degrade stable A3H is determined by the two amino acid residues at positions 39 and 48 (Fig 1B) [18–20]. Consistent with a prior study [20], hyper HIV-1 fully counteracted the anti-viral activity mediated by A3H-II, whereas hypo Vif was not able to counteract A3H-II (Fig 1B). In the absence of A3H-II, the infectivity of both of these HIV-1 molecular clones is similar (Fig 1B).

To investigate the impact of endogenous A3H on HIV-1 replication *in vivo*, a series of hyper versus hypo Vif competition experiments was conducted in humanized mice reconstituted with stable A3H-expressing HSCs. The first experiment used eight humanized mice, which were heterozygous for stable *A3H* haplotypes (S1 Table): two out of the eight had blood cell compartments reconstituted with haplotypes III and V cells, and the other six mice expressed haplotypes I and II (S1 Table). Next, these eight mice were intraperitoneally co-inoculated with equal amounts of hyper and hypo viruses (1,500 TCID_50_ each; Fig 1C), and the amount of viral RNA in the plasma and the level of human CD4^+^ T cells in the peripheral blood (PB) were routinely analyzed for 6 weeks post-infection (wpi). HIV-1 efficiently expanded in the humanized mice, as observed in our previous studies [11,12,22–24], and the level of peripheral CD4^+^ T cells was significantly reduced compared to mock-infected mice (Fig 1D). At 6 wpi, viral RNA was extracted from the plasma of infected mice and the sequences of the *vif* gene were analyzed. As anticipated, hyper *vif* and its derivatives were able to outcompete hypo *vif* virus in mice expressing stable A3H (73.1% ± 7.7% [286/391] in Fig 1E; see also S2 Table). However, hypo *vif*-related sequences were the majority in infected mouse no. 5 and were still present at significant levels in all animals (no. 5, 67.3% [35/52] to no. 1, 7.9% [3/38]; Fig 1E & S2 Table). These results raised the possibility that these hypo *vif* viruses may have adapted *in vivo* and gained a better ability to counteract stable A3H during viral replication. To address this idea, we subcloned the 13 hypo *vif-*related open reading frames (ORFs) into the expression plasmid and evaluated their anti-stable A3H activity using *in vitro* cell culture system. As shown in Fig 1F, adapted Vif proteins with V39F/N48H (26 clones from 4 mice), V39F/N48H/D113N (1 clone from 1 mouse) and V39F/N48H/L148S (1 clone from 1 mouse) mutations, degraded A3H-II and impaired the A3H-II packaging into the released viral particles. Additionally, the HIV-1 restriction capacity of A3H-II was significantly counteracted by these 3 adapted hypo Vif derivatives as evidenced by hyper Vif levels of infectivity (Fig 1G). We verified that these 3 hypo Vif derivatives as well as parental hypo Vif were active in counteracting other HIV-1 relevant A3s such as A3D, A3F and A3G (S1 Fig). Together, the sequencing results and the tests of the functionality of the adapted hypo Vif proteins indicated that 80.3% ± 4.8% (314/391) of the *vif* sequences in the plasma of infected stable A3H mice are able to counteract stable A3H (Fig 1E–1G & S2 Table). These findings indicate that the ability to antagonize stable A3H is required for efficient HIV-1 replication in humanized mice.

### Intermediate A3H-I does not elicit selective pressure on HIV-1 replication in humanized mice

**Fig 2:**
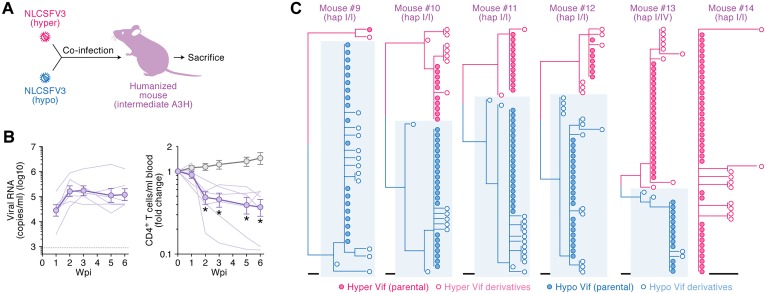
Dynamics of hyper/hypo HIV-1 infection in intermediate A3H humanized mice. (**A, B**) HIV-1 infection in humanized mice. (**A**) A schematic of co-inoculation of hyper and hypo HIV-1s into intermediate A3H humanized mice. (**B**) Hyper and hypo viruses containing 2.5 ng of p24 antigen each (5 ng in total; n = 6) or RPMI1640 (n = 12; for mock infection) were inoculated into humanized mice. The amount of viral RNA in plasma (left) and the level of peripheral CD4^+^ T cells (CD45^+^ CD3^+^ CD4^+^ cells) (right) were analyzed at 0, 1, 2, 3, 5, and 6 wpi as described in Materials and Methods. The averages are shown in circles with SEMs, and the values from each mouse are shown by line. X-axes, wpi. In the left panel, horizontal broken line indicates detection limit (800 copies/ml plasma). In the right panel, *P < 0.05 versus mock-infected mice by Mann-Whitney U test. (**C**) Phylogenetic trees of *vif* sequence. Viral *vif* sequences in the plasma of infected mice at 6 wpi were analyzed as described in Materials and Methods. Results of each infected mouse (mice #9–14) are respectively shown. Blue shadow indicates hypo *vif* derivatives. Scale bar represents one nucleotide substitution.

We next investigated whether HIV-1 undergoes selection as a result of pressure from A3H-I *in vivo*. Six humanized mice were reconstituted with HSCs from three individual donors. Five out of the six mice were homozygotes for A3H-I and one mouse was heterozygous for A3H haplotypes I and VI (S1 Table). These six mice were co-inoculated with hyper and hypo HIV-1s (Fig 2A). All mice exhibited a high level of viremia and a declined level of peripheral CD4^+^ T cells (Fig 2B). We then analyzed the *vif* sequences in the plasma of these six infected mice at 6 wpi. In contrast to the observations from animals with at least one copy of stable A3H (hap II or V; Fig 1E), the proportion of hyper/hypo *vif* sequences varied in each infected mouse, and no obvious replication biases were observed (Fig 2C & S3 Table). On average, the percentage of hyper and hypo *vif-*derived sequence were similar, 46.5% ± 13.7% (127/273) and, 53.5% ± 13.7% (146/273), respectively (S4 Table).

To assess whether *A3H* genotype affects the expression level of other HIV-1 relevant *A3* genes, we analyzed the expression levels of *A3D*, *A3F* and *A3G* in the splenic CD4^+^ T cells of humanized mice. Consistent with our previous studies with primary CD4^+^ T cells *ex vivo* and with humanized mice [24,25], the mRNA expression levels of these *A3* genes in HIV-1-infected mice were significantly higher than those in mock-infected mice (S2A Fig). The expression levels of these *A3* genes were comparable between the humanized mice expressing stable A3H and intermediate A3H (S2B Fig), indicating that the *A3H* genotype is not associated with the expression levels of other HIV-1 relevant *A3* genes.

In addition to *A3H*, single nucleotide variants (SNVs) in human *A3G* [26–28], *A3F* [29,30] and *A3D* [31] have been reported. The variants of A3G [28] and A3D [31] are degraded efficiently by HIV-1 Vif and are therefore unlikely to play significant roles *in vivo*. In contrast, An *et al*. have recently reported that an SNV of A3F, V231I, confers partial resistance to Vif-mediated degradation by certain strains of HIV-1 [30]. To address the possibility that A3F V231I mutant affects viral growth and the sensitivity to hyper/hypo HIV-1, we assessed the genomic sequences of *A3F*. However, this *A3F* SNV was not detected in the human cells used in our studies (data not shown). Altogether, these findings suggest that no specific selective pressure is elicited against either hyper or hypo HIV-1 in intermediate A3H humanized mice and that virus expansion is occurring in a stochastic manner.

### The ability to counteract stable A3H is acquired *de novo* during viral replication *in vivo*

**Fig 3:**
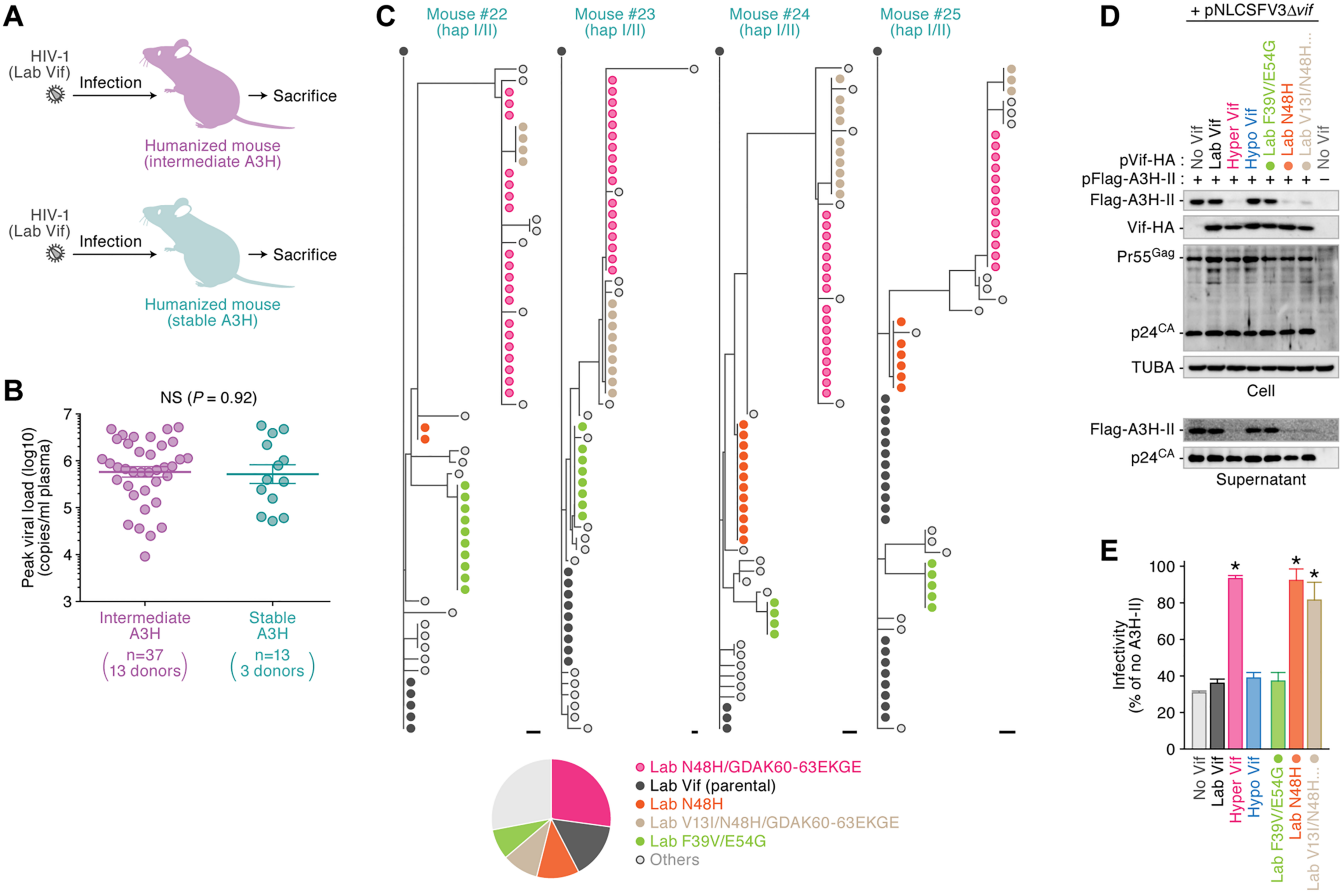
*De novo* emergence of hyper Vif in infected humanized mice with stable A3H. (**A, B**) HIV-1 infection in humanized mice. (**C**) A schematic of HIV-1 infection (strains AD8, JRCSF, NLCSFV3 and NL4-3) into the humanized mice with stable or intermediate A3H. (b) Peak VL of infected mice with intermediate or stable A3H. The values of peak VLs of the infected mice with intermediate A3H (n = 37 with 13 individual donors) and stable A3H (n = 13 with 3 individual donors) are summarized. Each dot represents the result from each mouse, and horizontal bars represent the averages with SEMs. NS, no statistic difference. (**C**) Phylogenetic trees of *vif* sequence. Viral *vif* sequences in the plasma of infected mice at 6 wpi were analyzed as described in Materials and Methods. Results of 4 infected mice with stable A3H (mice #22–25) are respectively shown. Each symbol represent identical sequence. Scale bar represents one nucleotide substitution. In the bottom panel, a pie chart represents the proportion of Vif derivatives detected in these infected mice. Raw data is shown in S4 Fig. (**D, E**) Evaluation of anti-stable A3H activity of Vif derivatives detected in infected mice. (**D**) Western blotting. The input of cell lysate was standardized to α-Tubulin (TUBA), and representative results are shown. (**E**) TZM-bl assay. The expression plasmids of the Vif derivatives were cotransfected with pNLCSFV3Δ*vif* and either with or without Flag-tagged A3H-II expression plasmid into HEK293T cells. The infectivity of released virus was determined by using TZM-bl cells, and the percentage of the value of "no A3H-II" is shown. *P < 0.05 versus "no Vif" by Student's *t* test. The assay was performed in triplicate. The data represents average with SD. In panels **C-E**, "Lab Vif" indicates NLCSFV3 Vif. In panels **D** and **E**, the symbols are identical to those in **Fig 3C**.

Co-infection studies revealed that hyper Vif HIV-1 dominates over hypo Vif virus in animals humanized with stable A3H expressing cells (Fig 1). These observations suggest that the stable A3H protein, which is expressed endogenously in human CD4^+^ T cells, exhibits a robust anti-viral effect and impairs the expansion of the viruses without full A3H counteraction abilities (i.e., V39 hypo Vif). We used three HIV-1 strains, NLCSFV3, JRCSF and AD8 to demonstrate that the Vif proteins of these viruses are unable to antagonize A3H-II (S3 Fig). We then inoculated these viruses into 50 humanized mice reconstructed from 16 HSC donors (Fig 3A). The genotyping PCR revealed that 13 out of the 16 HSC donors encode A3H-I, and 3 donors possessed one stable A3H allele (S5 Table). Based on *A3H* haplotypes, these infected mice were classified into two groups, intermediate A3H (n = 37) and stable A3H (n = 13), and the level of peak viral load in each group was compared. As shown in Fig 3B, surprisingly, the peak viral load was comparable between intermediate A3H mice and stable A3H mice (*P* = 0.92 by Mann-Whitney U test). Because certain hypo *vif* derivatives acquired anti-stable A3H activity in the stable A3H mice co-inoculated with hyper/hypo HIV-1 (Fig 1E–1G), we hypothesized that these viruses acquired *de novo* resistance to stable A3H *in vivo* (strains NLCSFV3, JRCSF, or AD8; Fig 3B). To test this hypothesis, we analyzed the *vif* sequences in the plasma of the 4 stable A3H mice infected with HIV-1 (strain NLCSFV3) at 6 wpi. Notably, some Vif sequences were commonly detected in the 4 stable A3H mice infected with NLCSFV3 (Fig 3C; the raw data and mutation matrix are shown in S4 Fig). To investigate whether these mutant variants acquired anti-stable A3H activity *de novo*, we prepared the expression plasmids of these Vif derivatives and conducted *in vitro* experiments using our cell culture system. As shown in Fig 3D & 3E, we detected 2 Vif variants, N48H and V13I/N48H/GDAK60-63EKGE, that are able to antagonize A3H-II at the level observed for hyper Vif. In summary, 59.7% ± 5.1% (145/243) of the *vif* sequences in the plasma acquired the ability to counteract stable A3H (S6 Table), but such mutants were not detected in the intermediate A3H mice infected, solely, with NLCSFV3 (S5 Fig & S7 Table). Taken together, these findings suggest that the ability of HIV-1 Vif to antagonize stable A3H is acquired *de novo* during viral expansion *in vivo*.

### Up-regulation of endogenous *A3H* expression by HIV-1 infection *in vivo*

**Fig 4:**
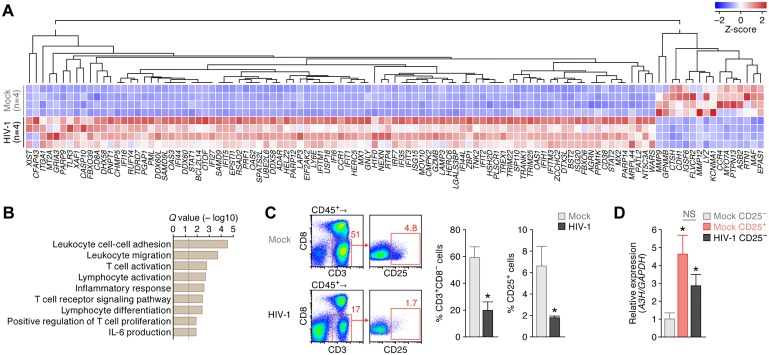
Immune activation and up-regulation of endogenous *A3H* expression in the human CD4^+^ T cells of infected humanized mice. (**A, B**) RNA-seq of the splenic human MNCs of infected humanized mice. (**A**) RNA-seq. Results of differentially expressed gene analysis from the splenic human MNCs of HIV-1-infected (n = 4) and mock-infected (n = 4) humanized mice are shown as a heatmap. (**B**) GSEA analysis. The procedure is described in Material and Method, and the top 50 annotations are listed in S8 Table. The vertical broken line indicates *Q* value = 0.05. (**C, D**) *A3H* expression in the human CD4^+^ T cells of infected mice. (**C**) Flow cytometry. Human MNCs were isolated from the spleen of HIV-1-infected (n = 6) and mock-infected (n = 6) humanized mice and analyzed the proportion of CD4^+^ T cells (CD45^+^ CD3^+^ CD8^−^ cells) and activated CD4^+^ T cells (CD45^+^ CD3^+^ CD8^−^ CD25^+^ cells) by flow cytometry. Representative dot plots (left), the percentage of CD3^+^ CD8^−^ cells in CD45^+^ cells (middle) and the percentage of CD25^+^ cells in CD3^+^ CD8^−^ cells (right) are respectively shown. *P < 0.05 versus mock-infected mice by Mann-Whitney U test. In panel **C**, the numbers on each dot plot indicates the percentage of gated cells. (**D**) Real-time RT-PCR of *A3H*. Activated CD4^+^ T cells (CD45^+^ CD3^+^ CD8^−^ CD25^+^ cells) and non-activated CD4^+^ T cells (CD45^+^ CD3^+^ CD8^−^ CD25^−^ cells) of mock-infected mice (n = 6 each) and CD25^−^ CD4^+^ T cells (CD45^+^ CD3^+^ CD8^−^ CD25^−^ cells) of HIV-1-infected mice (n = 6) were sorted using FACSJazz (see also S7 Fig). The mRNA expression level of *A3H* in each population was analyzed by real-time RT-PCR as described in Materials and Methods. The value of CD25^−^ CD4^+^ T cells of mock-infected mice is set as 1. *P < 0.05 versus CD25^−^ CD4^+^ T cells of mock-infected mice by Mann-Whitney U test. NS, no statistic difference. Note that CD25^+^ CD4^+^ T cells of infected mice were not available because this fraction was severely depleted (panel **C**).

We previously reported that endogenous *A3H* mRNA expression levels in primary human CD4^+^ T cells are significantly lower than those of anti-viral *A3* genes such as *A3D*, *A3F*, and *A3G*, and that activation and/or infection stimuli induces higher *A3H* expression [25,32]. In agreement with these prior works, the activation stimuli driven by anti-CD3/CD28 antibodies induced the expression of CD25, a marker of activated human CD4^+^ T cells (S6A Fig), and also *A3H* mRNA expression levels (*P* = 0.010 by paired *t* test, S6B Fig). However, it should be noted that human CD4^+^ T cells in humanized mice [12] and human PB [33] are less activated (S6C Fig) and in a quiescent state. In this regard, previous studies reported that HIV-1 infection induces the activation of CD4^+^ T cells of infected individuals [34,35]. Therefore, we hypothesized that HIV-1 infection induced CD4^+^ T-cell activation and augmented *A3H* expression in humanized mice, and this resulted in robust anti-viral effect by endogenous A3H (Figs 1 & 3). To investigate the immune activation status in detail, we performed RNA sequencing (RNA-seq). Human mononuclear cells (MNCs) were isolated from the spleen of 4 HIV-1-infected mice and 4 mock-infected mice at 6 wpi, and RNA-seq analyses were conducted. As shown in Fig 4A, 93 genes were significantly up-regulated by HIV-1 infection, whereas 16 genes were down-regulated. Parametric gene set enrichment analysis (GSEA) revealed that the genes associated with T-cell/lymphocyte activation, inflammatory response, and positive regulation of T cell activation were significantly up-regulated in the human MNCs of HIV-1-infected mice (Fig 4B; the GESA result is listed in S8 Table). In addition, various interferon-stimulated genes such as *RSAD2* (encoding Viperin), *DDX58* (encoding RIG-I), *EIF2AK2* (encoding PKR), *MX1*, *ISG15*, *MOV10* and *BST2* (encoding tetherin) were up-regulated in HIV-1-infected mice (Fig 4A). As observed in infected patients [34,35], our findings suggest that HIV-1 infection triggers immune activation in humanized mouse model.

We then addressed the possibility that the immune activation caused by HIV-1 infection (Fig 4A & 4B) leads to the up-regulation of *A3H* in humanized mice. As shown in Fig 4C, the proportion of the splenic CD4^+^ T cells (CD3^+^ CD8^−^ cells) of infected mice was significantly lower than that of uninfected mice, and particularly, CD25^+^ activated CD4^+^ T cells were severely depleted by HIV-1 infection (*P* = 0.0039 versus mock infection). Consistent with our previous findings [12], HIV-1 infection led to the severe depletion of activated CD4^+^ T cells in humanized mice.

Next, we sorted the fractions of non-activated CD4^+^ T cells (CD45^+^ CD3^+^ CD8^−^ CD25^−^ cells) and activated CD4^+^ T cells (CD45^+^ CD3^+^ CD8^−^ CD25^+^ cells) of mock-infected mice (S7 Fig) and analyzed the mRNA expression level of *A3H* in each population by real-time RT-PCR. In mock-infected mice, *A3H* expression in the activated CD4^+^ T cells was significantly higher than that in non-activated cells (*P* = 0.0090 by Mann-Whitney U test; Fig 4D). This finding further suggests that the CD4^+^ T-cell activation augments *A3H* expression, as observed in *in vitro* experiments (S6B Fig) and in our previous reports [25,32]. Because CD25^+^ CD4^+^ T cells were severely depleted in infected mice (Fig 4C), we sorted only the fraction of CD25^−^ CD4^+^ T cells (CD45^+^ CD3^+^ CD8^−^ CD25^−^ cells) of HIV-1-infected mice for real-time RT-PCR. Interestingly, the *A3H* expression level in the CD25^−^ CD4^+^ T cells of HIV-1-infected humanized mice was significantly higher than that of CD25^−^ CD4^+^ T cells of uninfected mice (*P* = 0.0062 by Mann-Whitney U test; Fig 4D). Altogether, these findings suggest that the immune activation triggered by HIV-1 infection augments *A3H* expression in CD4^+^ T cells of infected humanized mice.

### *A3H* haplotype may influence HIV-1 spread in human population

**Fig 5:**
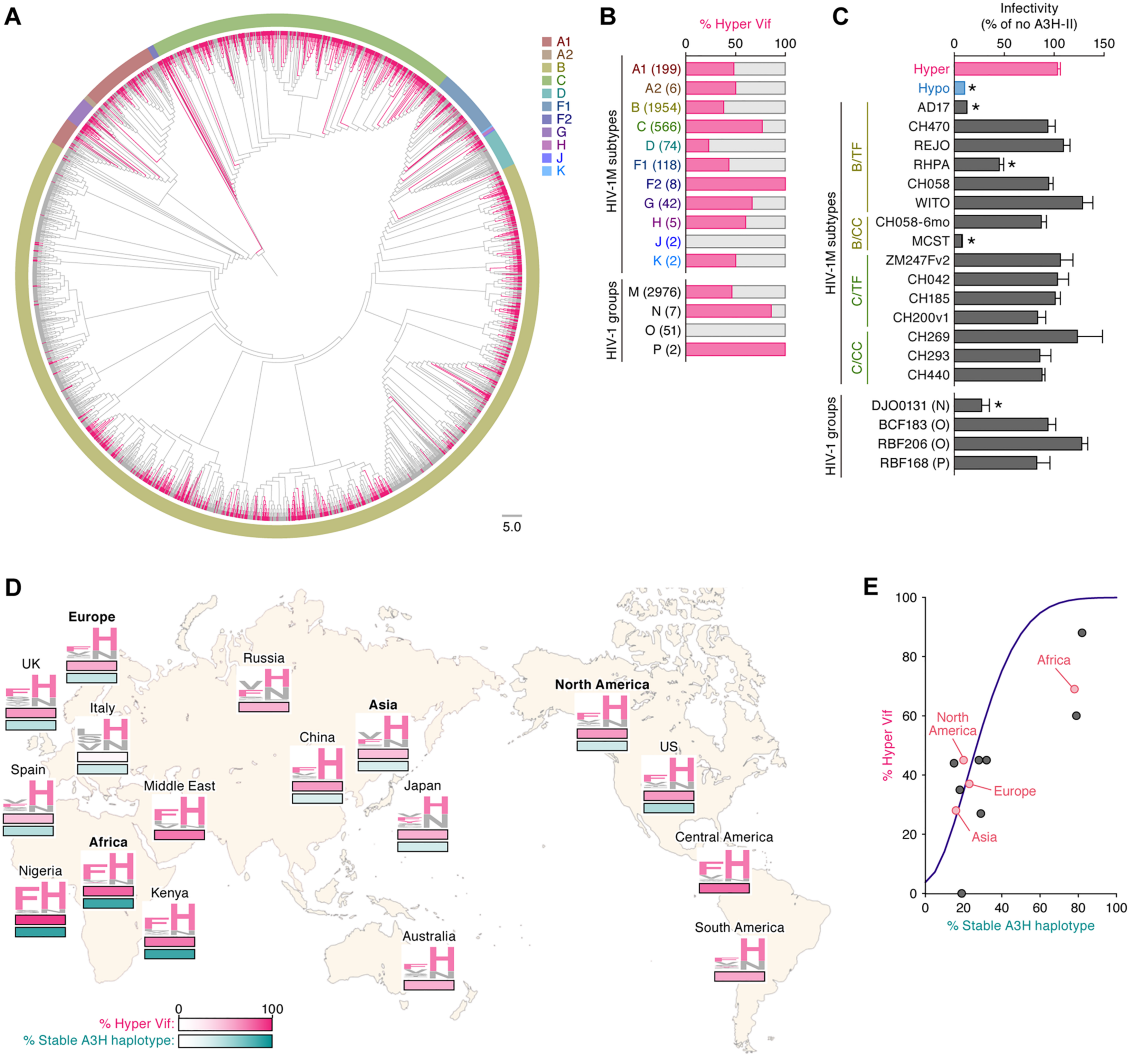
Dynamics of hyper/hypo HIV-1 dissemination in human population. (**A**) A phylogenetic tree of Vif. The Vif sequences were extracted from HIV-1 sequence database (https://www.hiv.lanl.gov/components/sequence/HIV/search/search.html) and the phylogenetic tree was constructed as described in Materials and Methods. The branches of hyper Vif sequences (i.e., F or Y in position 39 and H in position 48) are indicated with pink. Each color surrounding the phylogenetic tree represents viral subtype (A1-K). Scale bar indicates 5.0 amino acid substitutions per site. (**B**) The percentage of hyper Vif sequences in each subtype and group. The sequences of HIV-1 Vif is classified into hyper and hypo Vif based on the amino acids positioned at 39 and 48 and the result is summarized. The numbers in parentheses represents the number of Vif sequences used. See also S10 Table. (**C**) TZM-bl assay. The IMCs (1,000 ng) were cotransfected either with or without Flag-tagged A3H-II expression plasmid (50 ng) into HEK293T cells. The infectivity of released virus was determined by using TZM-bl cells, and the percentage of the value of "no A3H-II" is shown. *P < 0.05 versus "hyper HIV-1" by Student's *t* test. The assay was performed in triplicate. The data represents average with SD. TF, transmitted/founder; CC, chronic control. See also S9 Table. (**D**) Distribution of hyper HIV-1 and individuals with stable A3H in the world. The percentages of hyper HIV-1 (pink, top) and stable A3H haplotype (green, bottom) in each region (Europe, Africa, Asia and North America; represented in bold) and country were obtained as described in Materials and Methods, and these two values are indicated by heatmap. The Vif amino acids at positions 39 and 48 are shown in logoplot, and the residues associated with hyper Vif (i.e., F or Y in position 39 and H in position 48) are represented in pink. Note that the information of the proportion of *A3H* haplotype is not available in Russia, Australia, Central America and South America. See also S11 Table. (**E**) Mathematical modeling of the dissemination of hyper HIV-1 in human population. The prevalence of hyper HIV-1 in the human population with different stable A3H proportion was simulated by the mathematical model (for the detail, see Materials and methods). The simulated prediction is shown with purple line. Red and Black dots indicate the results from respective regions and countries (see also S9 Fig & S12 Table).

Finally, we addressed how hyper and hypo HIV-1 sequences circulate in the human population. The HIV-1 Vif sequences were obtained from the Los Alamos National Laboratory HIV-1 sequence database (https://www.hiv.lanl.gov/components/sequence/HIV/search/search.html). Fig 5A shows a phylogenetic tree of Vif sequences of HIV-1 group M (n = 2,976), which is a pandemic strain worldwide. The phylogenetic tree indicates that Vif sequences cluster based on subtype (Fig 5A). Interestingly, the sequences of hyper Vif (here we defined "hyper Vif" as a sequence that possesses F or Y at position 39 and H at position 48) scattered in this tree and did not form a unique cluster (Fig 5A). Additionally, the percentage of hyper Vif varied in each subtype (Fig 5B), suggesting that hyper and hypo Vif mutually swap in human population.

To evaluate the counteracting ability of HIV-1 clinical isolates (group M) against stable A3H, we used 15 infectious molecular clones (IMCs): 8 subtype B and 7 subtype C; 10 transmitted/founder (TF) viruses and 5 chronic control (CC) viruses. As shown in Fig 5C, the infectivity of the 3 IMCs (strains AD17, RHPA and MCST) was significantly suppressed by A3H-II with statistical differences, suggesting that these viruses have established new infection as TF viruses in individuals without anti-stable A3H activity. In contrast, other 12 IMCs overcame A3H-II-mediated restriction (Fig 5C), suggesting that these viruses exist in human population as hyper HIV-1. Importantly, the anti-stable A3H ability of these IMCs corresponded well to the amino acid residues positioned at 39 and 48 (S9 Table). These findings suggest that anti-stable A3H ability is not a necessary requirement for certain viruses circulating within individuals.

We then assessed the anti-stable A3H ability of non-pandemic HIV-1 groups N, O, and P. As shown in Fig 5B, the proportion of hyper Vif sequences varied in each group. In particular, HIV-1 group O strains (n = 51) did not encode a hyper *vif* sequences. However, the cell-based experiments demonstrated that the IMCs of group O (strains BCF183 and RBF206) overcame A3H-II-mediated anti-viral effect (Fig 5C). The IMC of HIV-1 group P (strain RBF168) also counteracted A3H-II, while that of HIV-1 group N (strain DJO0131) did not (Fig 5C). Interestingly, in contrast to the results of HIV-1 group M, the anti-stable A3H ability of HIV-1 group O Vif was not governed by the two residues at positions 39 and 48 (S9 Table) These findings suggest that other residues than those positioned at 39 and 48 determine the ability of Vif proteins of HIV-1 group O to counteract stable A3H.

Furthermore, we assessed the correlation between the frequency of hyper HIV-1 and the proportion of the individuals harboring stable *A3H* haplotype worldwide. The HIV-1 Vif sequences were obtained from HIV-1 sequence database (S10 Table), and the information of *A3H* haplotype was obtained from the 1000 Genomes Project (http://www.internationalgenome.org) [36] (S11 Table). As shown in Fig 5A & 5B, the Vif sequences are highly diversified and the logoplot (S8 Fig) further indicated that the amino acids at position 39 and 48 were not highly conserved when compared to the YRHHY motif, which is essential for A3G degradation [24,37]. Additionally, consistent with previous reports [14,15,20], both the percentage of hyper Vif and the proportion of stable *A3H* haplotype were highest in Africa, particularly in Nigeria (Fig 5D & S12 Table), and these two parameters were correlated each other with statistical significance (Spearman's *r* = 0.720, *P* = 0.017 by Spearman rank correlation test; S9 Fig). To further investigate the relationship between hyper HIV-1 and stable *A3H* haplotype, we conducted a mathematical simulation. As shown in Fig 5E, the frequency of hyper HIV-1 increased dependent on the proportion of the people harboring stable *A3H* haplotype. Taken together, our analyses at a human population level suggest that stable A3H elicits a selective pressure against HIV-1, and that HIV-1 overcomes stable A3H-mediated anti-viral immunity by acquiring the ability to counteract stable A3H.

## Discussion

In this study, we used a humanized mouse model to show that HIV-1 infection induces immune activation and augments the expression of endogenous *A3H* in human CD4^+^ T cells (Fig 4). We also showed that the ability of HIV-1 Vif to counteract stable A3H-mediated anti-viral effect is crucial for efficient viral expansion *in vivo* when endogenous A3H is expressed stably (Figs 1 & 3). In contrast, the ability of HIV-1 Vif to counteract stable A3H is dispensable when stable A3H is absent *in vivo* (Fig 2). Furthermore, we addressed the significance of the stable A3H-mediated anti-viral effect on HIV-1 dissemination in human populations using molecular phylogenetic analysis and mathematical modeling. The occurrence of hyper Vif variants and stable A3H haplotypes correlates worldwide, suggesting that the ability of Vif to antagonize stable A3H was acquired during viral spread throughout the human population (Fig 5). These findings suggest that the A3H polymorphism influences HIV-1 dissemination at individual and population levels.

In the stable A3H humanized mice co-inoculated with hyper and hypo HIV-1 infectious clones, several hypo Vif viruses acquired V39F and N48H changes, which resulted in the gain-of-function to counteract A3H-II (Fig 1E–1G). Given that these two amino acids are identical to those in hyper Vif, the emergence of the hypo Vif derivatives, which can potently antagonize A3H-II in the hyper/hypo HIV-1 co-inoculated stable A3H mice (Fig 1), may be due to the recombination between hypo and hyper Vif sequences. However, the results shown in Fig 3 argue against this possibility. We demonstrated that some Vif sequences with the ability to antagonize stable A3H emerge during viral replication in the stable A3H mice within only 6 weeks. In contrast, in the intermediate A3H mice co-inoculated with hyper and hypo HIV-1 clones, the hyper or hypo Vif viruses expanded randomly with no evidence of selection on *vif* (Fig 2). These findings suggest that the stable A3H, which is endogenously expressed in CD4^+^ T cells, has a robust anti-viral activity *in vivo* and that it is feasible for Vif to acquire the counteracting ability against stable A3H *de novo*. We favor a model in which the starting hypo Vif virus is constrained evolutionarily, likely by needing to counteract A3D, A3F, and A3G, and that *de novo* (not recombination mediated) amino acid substitutions at positions 39 and 48 provide the most efficient route to optimize anti-A3H activity. Moreover, it is important to note that all the stable A3H humanized mice used in this study were heterozygous for A3H stability (S1 & S5 Tables). It appears that an allele of stable A3H is sufficient to induce a robust selective pressure against HIV-1.

In sharp contrast to the findings in the stable A3H mice co-inoculated with hyper and hypo HIV-1s (Fig 1), hyper HIV-1 was not commonly selected in the intermediate A3H mice co-inoculated with hyper and hypo HIV-1 clones (Fig 2). Also, *de novo* emergence of hyper Vif was not detected in the intermediate A3H mice infected with NLCSFV3 (S5 Fig). On the other hand, we recently showed that the intermediate A3H (A3H-I) is enzymatically active and contributes to breast and lung cancer mutagenesis despite being expressed at lower levels compared to its stable A3H counterpart [17]. These findings suggest that A3H-I, which is endogenously expressed in human CD4^+^ T cells, is not sufficient to impose selective pressure on HIV-1 replication *in vivo*.

Here we detected the emergence of Vif sequences that acquired the ability to antagonize stable A3H (Figs 1 & 3). In contrast, in the humanized mice infected with a *vif-*mutated HIV-1 (designated 4A HIV-1), which is sensitive to A3D and A3F, we have previously demonstrated that Vif sequences with the ability to antagonize A3D and A3F do not emerge [24]. We confirmed the absence of Vif revertants in the plasma of two 4A-HIV-1 infected mice infected at 6 wpi (S10 Fig). These observations suggest that HIV-1 is able to overcome the restriction mediated by stable A3H but not by A3D and A3F during viral replication *in vivo*. Two nonexclusive models may explain the observed differences. One possibility is that it might be more feasible for Vif to overcome stable A3H-mediated restriction than A3D/A3F because the anti-viral activity of endogenous stable A3H is lower than those of endogenous A3D and A3F. However, at least four previous studies have demonstrated that the anti-HIV-1 activity of stable A3H (haplotype II) is similar to that of A3F and is higher than that of A3D [18,19,25,38] and argue against this possibility. In addition, it should be noted that the endogenous expression levels of the respective *A3* genes in primary human CD4^+^ T cells are different from each other. Indeed, Refsland *et al*. have revealed that endogenous expression levels of *A3D* and *A3F* mRNAs are higher than that of *A3H* in primary CD4^+^ T cells [32]. Another possibility is the number of amino acids responsible for A3 counteraction: only two amino acids at positions 39 and 48 are responsible for counteracting stable A3H [18–20], while there are four that are responsible for counteracting A3D and A3F (known as DRMR motif at position 14–17) [37,39].

The emergence of Vif revertants harboring the ability to counteract stable A3H is reminiscent of the observations that the sub-optimal drug concentrations facilitate the emergence of drug-resistant viruses in infected patients [40,41]. In fact, it appears difficult for Vif to acquire the ability to counteract A3F and A3G *de novo* during viral replication in cell cultures [42,43] and a humanized mouse model [11,24]. In contrast, previous studies have successfully selected the viruses that acquired the ability to counteract stable A3H in the *in vitro* culture infection experiments using the human CD4^+^ T cell lines such as MT-4 cells [18] and SupT11 cells [20] that ectopically express A3H-II. Here we demonstrated that HIV-1 infection induces immune activation in humanized mice, as observed in infected individuals [34,35], and augments the expression of endogenous *A3H* in the human CD4^+^ T cells of infected mice (Fig 4). But still, the anti-HIV-1 activity of endogenous stable A3H is not sufficient to control viral expansion *in vivo*, and therefore, Vif may easily acquire the ability to counteract the restrictive activity of endogenous A3H.

Our findings in infected humanized mice revealed that hyper HIV-1 is predominantly selected in the mice expressing stable A3H (80.3% ± 4.8%; S2 Table), while the viruses replicated in the mice with intermediate A3H were selected stochastically (46.5% ± 13.7%; S4 Table). We also demonstrated the *de novo* emergence of hyper HIV-1 in the stable A3H mice infected with NLCSFV3 (59.7% ± 5.1%; S6 Table). Based on these findings and numerical parameters, we investigated the dynamic effect of A3H haplotypes on HIV-1 epidemic in the human population through molecular phylogenetic and mathematical modeling and revealed that the occurrence of hyper Vif and stable A3H variants are correlated positively in the human population (Fig 5). This suggests that stable A3H may not just provide an intrinsic immunity at the level of individual patients, as elaborated here in humanized mice, but it may also function to control the dissemination of hypo HIV-1 isolates in the human population [44,45].

## Materials and methods

### Ethics statement

All procedures including animal studies were conducted following the guidelines for the Care and Use of Laboratory Animals of the Ministry of Education, Culture, Sports, Science and Technology, Japan. The authors received approval from the Institutional Animal Care and Use Committees (IACUC)/ethics committee of the institutional review board of Kyoto University (protocol number D15-08). All protocols involving human subjects were reviewed and approved by the Kyoto University institutional review board. All human subjects were provided written informed consent from adults.

### Humanized mice

NOG mice (NOD/SCID/*Il2r* KO mice) [46] were obtained from the Central Institute for Experimental Animals (Kawasaki, Kanagawa, Japan). The mice were maintained under specific-pathogen-free conditions and were handled in accordance with the regulation of the IACUC/ethics committee of Kyoto University. Human CD34^+^ hematopoietic stem cells (HSCs) were isolated from human fetal liver as previously described [47]. The humanized mouse model (NOG-hCD34 mouse) was constructed as previously described [11,22,23,48–50]. In the experiments shown in Figs 1 & 2, 14 newborn (aged 0 to 2 days) NOG mice from 7 litters were irradiated with X-ray (10 cGy per mouse) using an RX-650 X-ray cabinet system (Faxitron X-ray Corporation) and were then intrahepatically injected with the human fetal liver-derived CD34^+^ cells (1.0 × 10^5^ to 2.3 × 10^5^ cells; 5 donors). A list of the humanized mice used in this study is summarized in S1 Table. In the experiments shown in Fig 3, the 35 NOG-hCD34 mice infected with HIV-1 were used in our previous studies [12,23,24] (Fig 3) and the 15 NOG-hCD34 mice were newly infected with HIV-1. These humanized mice were constructed using 16 independent HSC donors with 29 NOG litters (summarized in S5 Table).

### Cell culture

HEK293T cells (a human embryonic kidney 293 T cell line; ATCC CRL-3216) and TZM-bl cells (obtained through the NIH AIDS Research and Reference Reagent Program) [51] were maintained in Dulbecco’s modified Eagle's medium (Sigma) containing FCS and antibiotics. Human peripheral CD4^+^ T cells were isolated human CD4^+^ T cell isolation kit (Miltenyi) according to the manufacturer’s protocol. These cells were activated with anti-CD3/anti-CD28 dynabeads (Thermo Fisher Scientific) and maintained in RPMI1640 (Sigma) containing FCS and antibiotics with human interleukin-2 (100 U/ml) as previously described [23].

### Virus preparation and infection

To construct the IMCs of hyper HIV-1 and hypo HIV-1 derivatives (based on a CCR5-tropic strain NLCSFV3 [21]), the hyper and hypo Vif variants of the HIVIIIB A200C proviral constructs [20] were digested with AgeI and EcoRI, then the resultant DNA fragment was inserted into the AgeI-EcoRI site of pNLCSFV3 [21]. The IMCs of HIV-1 strains JRCSF [52] and AD8 [53] were also used. The two *vif-*mutated derivatives based on pNLCSFV3, *vif-*deleted virus (pNLCSFV3Δ*vif*) and DRMR/AAAA-mutated virus (4A HIV-1), are constructed in our previous study [11,24]. The IMCs of transmitted/founder (TF) and chronic control (CC) viruses as well as those of HIV-1 groups N (strain DJO0131), O (strains BCF183 and RBF206) and P (strain RBF168) (Fig 5C) were obtained kindly provided by Drs. Beatrice H. Hahn (University of Pennsylvania, USA) and Frank Kirchhoff (Ulm University Medical Center, Germany).

To prepare virus solutions of hyper and hypo HIV-1s, 30 μg of each IMC was transfected into HEK293T cells according to calcium-phosphate method as previously described [11,12,23,24]. At 48 h posttransfection, the culture supernatant was harvested, centrifuged, and then filtered through a 0.45-μm filter (Millipore) to obtain the virus solution. The amount of viral particles was quantified using an HIV-1 p24 (Gag) antigen ELISA kit (Zeptometrix). Virus solutions of hyper and hypo HIV-1 clones (containing 2.5 ng of Gag antigen each) were intraperitoneally co-inoculated into NOG-hCD34 mice. RPMI 1640 was used for mock infection.

### PB collection, MNC isolation and quantification of HIV-1 RNA in plasma

PB and plasma were routinely collected as previously described [11,12,22–24]. The mice were euthanized at 6 wpi with anesthesia and the spleen was crushed, rubbed, and suspended as previously described [11,12,22–24]. To obtain splenic human MNCs, the splenic cell suspension was separated using Ficoll-Paque (Pharmacia) as previously described [11,12,22–24]. The amount of HIV-1 RNA in 50 μl plasma was quantified by Bio Medical Laboratories, Inc. (the detection limit of HIV-1 RNA is 800 copies/ml).

### Genotyping PCR

In the experiments shown in Figs 1 & 2, genomic DNA was extracted from the PB of NOG-hCD34 mice using a DNeasy Blood & Tissue kit (Qiagen) as previously described [24]. In the experiments shown in Fig 3, genomic DNA was extracted from the splenic MNCs of NOG-hCD34 mice in the same procedure. Genotyping PCR of *A3H* was performed using PfuUltra High Fidelity DNA polymerase (Agilent) according to the manufacturer’s protocol, and the following primers were used: Exon2_Fwd, 5'-GAA ACA CGA TGG CTC TGT TAA CAG CC-3'; Exon3_Rev, 5'-CGG GGG TTT GCA CTC TTA T-3'; Exon4_Fwd, 5'-AGG AAG GAA GGA TTG TGG CTC A-3'; Exon4_Rev, 5'-GAG TCC TCA TGC TCA GCA CA-3' (see also Fig 1A). For genotyping PCR of *A3F*, the following primers were used: A3F_exon5_8822_Fwd. 5'-GGT CTC TGC ATT GGG GTT TC-3'; A3F_exon5_9069_Rev: 5'-TGC ATT CCT AGC TGC TTA GC-3'. The resulting DNA fragments were directly sequenced, and, if needed, were cloned using a zero blunt TOPO PCR cloning kit (Thermo Fisher Scientific). The sequence was analyzed with Sequencher v5.1 software (Gene Codes Corporation).

### Flow cytometry, hematometry and cell sorting

Flow cytometry was performed with FACS Calibur (BD Biosciences) and FACSJazz (BD Biosciences) as previously described [11,12,22–24], and the obtained data were analysed with Cell Quest software (BD Biosciences) and FlowJo software (Tree Star, Inc.). For flow cytometry analysis, anti-CD45-PE (HI30; Biolegend), anti-CD3-APC-Cy7 (HIT3a; Biolegend), anti-CD4-APC (RPA-T4; Biolegend), anti-CD25-APC (BC96; eBioscience), and anti-Ki67-PE (B56; BD Biosciences) antibodies were used. Hematometry was performed with a Celltac α MEK-6450 (Nihon Kohden Co.) as previously described [11,12,23,24,49]. Live cell sorting was performed using FACSJazz (BD Biosciences) according to the manufacture's procedure. The purity of each population was >94% (see also S7 Fig).

### Transfection, TZM-bl assay and Western blotting

Transfection, the TZM-bl assay and Western blotting were performed as previously described [11,12,23,24]. Briefly, in the experiments shown in Fig 1B & S3 Fig, HEK293 cells were cotransfected with an expression plasmid for flag-tagged A3H-II (0, 25, 50 and 100 ng) and the indicated IMCs (1 μg). In the experiments shown in Figs 1F, 1G, 3D & 3E, HEK293 cells were co- cotransfected with an expression plasmid for flag-tagged A3H-II (10 ng), pNLCSFV3Δ*vif* (500 ng) and an expression plasmid for the indicated Vif tagged with HA (500 ng). In the experiments shown in Fig 5C, HEK293 cells were cotransfected with an expression plasmid for flag-tagged A3H-II (50 ng) and the indicated IMCs (1 μg). In the experiments shown in S1 Fig, HEK293 cells were cotransfected with an expression plasmid for flag-tagged A3D (50 ng), A3F (10 ng) or A3G (10 ng), pNLCSFV3Δ*vif* (500 ng) and an expression plasmid for the indicated Vif tagged with HA (500 ng). For Western blotting, anti-Flag antibody (M2; Sigma), anti-HA antibody (3F10; Roche), anti-p24 antiserum (ViroStat), and anti-α-tubulin (TUBA) antibody (DM1A; Sigma) were used.

### RT-PCR for *vif* cloning

RT-PCR was performed as previously described [24]. Briefly, viral RNA was extracted from the plasma of infected mice at 6 wpi using a QIAamp viral RNA mini kit (Qiagen), and cDNA was prepared as previously described [24]. RT-PCR was performed using PrimeSTAR GXL DNA polymerase according to the manufacturer’s protocol, and the following primers used are used: Vif-Fwd, 5'-GTT TGG AAA GGA CCA GCA AA-3'; Vif-Rev, 5'-GCC CAA GTA TCC CCG TAA GT-3'. The resulting DNA fragments were cloned using a zero blunt TOPO PCR cloning kit (Thermo Fisher Scientific), and the sequence was analyzed with Sequencher v5.1 software (Gene Codes Corporation).

### Molecular phylogenetic

The *vif* ORF sequences (Figs 1E, 2C & 3C) were aligned by using MUSCLE [54] implemented in MEGA 6 software [55]. ML phylogenetic trees were constructed using MEGA 5.1 software [55]. The Vif sequences (Fig 5A and 5B & S10 Table; one sequence per patient) were extracted from Los Alamos National Laboratory HIV-1 sequence database (https://www.hiv.lanl.gov/components/sequence/HIV/search/search.html). These sequences were aligned and the phylogenetic tree was constructed as described above.

### Plasmid construction

A series of HA-tagged Vif expression plasmids are based on pDON-AI (Takara) and are constructed in our previous study [24]. To prepare the expression plasmids of Vif derivatives (Figs 1F, 1G, 3D & 3E), the pCRII-blunt-TOPO containing *vif* ORFs were digested with EcoRI and blunted. The resultant DNA fragments containing *vif* ORF were subcloned into the HpaI site of pDON-AI (Takara).

### RNA-seq and data mining

Human MNCs were isolated from the spleen of humanized mice as described above and RNA was extracted using QIAamp RNA Blood Mini kit (Qiagen) as described above [11,23,24]. RNA-seq analysis was conducted in Medical & Biological Laboratories, co (Nagoya, Japan). The obtained raw sequence data (.fastq files) were mapped to the human reference genome (NCBI hg19) by Bowtie2 version 2.2.5 [56], followed by spliced junction detection by Tophat2 version 2.1.0 [57]. Several R (versions 3.1.1) and Bioconductor packages were used to further process the gene expression data. Read count data for each sample were extracted by package ‘Rsubread’ [58]. The obtained raw read count data were then normalized by applying repeated edgeR normalization defined in package ‘TCC’ [59]. The normalized read count data were classified into two groups according to infection status (HIV-1 infected, or uninfected as control). The expression data were analyzed to detect differentially expression genes by package edgeR [60]. Top-ranked genes were selected as differentially expressed genes (DEGs) with the following threshold values: False Discovery Rate (FDR) less than 0.001 calculated by the Benjamini-Hochberg method [61], and more than twice up-regulated or less than half down-regulated normalized gene expressions compared with the control (see Fig 4B & S8 Table). DEGs were then used to obtain enriched biological functions by a parametric gene set enrichment analysis by using package ‘gage’ [62]. The method defined in ‘gage’ enabled to extract gene ontology terms associated with up-regulated DEGs. Finally, a distance matrix was calculated from the expression data for DEGs based on the correlation distance [63], and the distance matrix was converted by the Z-transformation defined in package ‘gplots’ to visualize the result with a heatmap (Fig 4A).

### Real-time RT-PCR

Real-time RT-PCR was performed as previously described [20,24] using CFX connect real-time system (Biorad) and the following primers: A3H-Fwd (RSH2757), 5'- AGC TGT GGC CAG AAG CAC-3' and A3H-Rev (RSH2758), 5'-CGG AAT GTT TCG GCT GTT-3'. *A3D*, *A3F*, *A3G* were amplified by using the primers reported previously [32], and the primers for *GAPDH* were purchased from Thermo Fisher Scientific.

### Database analysis

The information of *A3H* haplotypes of individuals was extracted from the 1000 Genomes Project (http://www.internationalgenome.org) [36]. We obtained the Phase 1 VCF (variant call format) data of 1092 individuals from all available human populations. From this phased variant dataset we extracted the information of 5 A3H SNPs 15, 18, 105, 121, and 178 and estimated the frequencies of *A3H* haplotypes for each population.

### Mathematical modeling and simulations

The following simple model describes the HIV-1 transmission among human population:

$$\frac{dS\left(t\right)}{dt}=b-dS\left(t\right)-\frac{\beta S\left(t\right)I\left(t\right)}{N\left(t\right)},\;\frac{dI\left(t\right)}{dt}=\frac{\beta S\left(t\right)I\left(t\right)}{N\left(t\right)}-\mu I\left(t\right),$$

where *S*(*t*) and *I*(*t*) represent the number of susceptible and infected individuals, respectively [64]. *N*(*t*) is the total population size at time *t*, and *N*(0) = *b*/*d* is the initial size. Susceptible individuals are born at rate *b* and removed at rate *d*, and infected individuals transmit HIV-1 at a rate *β* during their infectious period of 1/*μ*. To describe the dissemination of hyper HIV-1 in the human population, we modified the above model as follows:

$$\frac{dS^{U}\left(t\right)}{dt}=b_{U}-dS^{U}\left(t\right)-\frac{\beta S^{U}\left(t\right)}{N\left(t\right)}\left\{I_{r}^{U}\left(t\right)+I_{o}^{U}\left(t\right)+I_{r}^{S}\left(t\right)+I_{o}^{S}\left(t\right)\right\},$$

$$\frac{dI_{r}^{U}\left(t\right)}{dt}=\frac{\beta S^{U}\left(t\right)}{N\left(t\right)}\left\{I_{r}^{U}\left(t\right)+I_{r}^{S}\left(t\right)\right\}-\mu I_{r}^{U}\left(t\right),$$

$$\frac{dI_{o}^{U}\left(t\right)}{dt}=\frac{\beta S^{U}\left(t\right)}{N\left(t\right)}\left\{I_{o}^{U}\left(t\right)+I_{o}^{S}\left(t\right)\right\}-\mu I_{o}^{U}\left(t\right),$$

$$\frac{dS^{S}\left(t\right)}{dt}=b_{S}-dS^{S}\left(t\right)-\frac{\beta S^{S}\left(t\right)}{N\left(t\right)}\left\{I_{r}^{U}\left(t\right)+I_{o}^{U}\left(t\right)+I_{r}^{S}\left(t\right)+I_{o}^{S}\left(t\right)\right\}$$

$$\frac{dI_{r}^{S}\left(t\right)}{dt}=\frac{\beta S^{S}\left(t\right)}{N\left(t\right)}\left\{I_{r}^{U}\left(t\right)+I_{r}^{S}\left(t\right)+fI_{o}^{S}\left(t\right)\right\}-\mu I_{r}^{S}\left(t\right),$$

$$\frac{dI_{o}^{S}\left(t\right)}{dt}=\frac{\beta S^{S}\left(t\right)}{N\left(t\right)}\left\{I_{o}^{U}\left(t\right)+\left(1-f\right)I_{o}^{S}\left(t\right)\right\}-\mu I_{o}^{S}\left(t\right).$$

The variable *S*^*U*^(*t*) is the number of susceptible individuals harboring unstable *A3H* haplotype, and $I_{r}^{U}\left(t\right)$ and $I_{o}^{U}\left(t\right)$ are the number of infected individuals with hyper and hypo HIV-1s, respectively. On the other hand, the variable *S*^*S*^(*t*) is the number of susceptible individuals harboring stable *A3H* haplotype, and $I_{r}^{S}\left(t\right)$ and $I_{o}^{S}\left(t\right)$ are the number of infected individuals with hyper and hypo HIV-1s, respectively. We assumed that the susceptible individuals harboring unstable and stable *A3H* haplotype are born at the rates *b*_*U*_ and *b*_*S*_ = *b* − *b*_*U*_, respectively. Furthermore, we considered that the fraction, *f*, of newly infected individuals harboring stable *A3H* haplotype with hypo HIV-1 become infected individuals with hyper HIV-1 because of adaptive evolution of hyper HIV-1 from hypo HIV-1 *in vivo*, as we observed in the stable A3H mice infected with NLCSFV3 (Fig 3 & S6 Table).

To investigate how the frequency of hyper HIV-1 at 100 years after the initial infection (i.e., $\left(I_{r}^{U}\left(100\right)+I_{r}^{S}\left(100\right)\right)/\left(I_{r}^{U}\left(100\right)+I_{r}^{S}\left(100\right)+I_{o}^{U}\left(100\right)+I_{o}^{S}\left(100\right)\right)$) is determined depend on the proportion of the people harboring stable *A3H* haplotype (i.e.,*S*^*S*^(0)/*N*(0) = (*b*_*S*_/*d*)/(*b*/*d*) = *b*_*s*_/*b*), we simulated the transmission dynamics of hyper and hypo HIV-1s among 1 million individuals for 0 < *b*_*s*_/*b* < 1 based on the above modified mathematical model. Here we simply fixed 1/*d* = 35 years (i.e., adults aged 15–49 years), which implies *b* = *dN*(0) = 2.86 × 10^4^ per year. As previously estimated in [65,66], we assumed that *β* = 4.53 per year, and 1/*μ* = 35 years corresponding to HIV-1-infected individuals with the mean set-point viral load of 3.2 × 10^4^ RNA copies/ml. The fraction, *f*, is fixed to be 0.60 in our simulations based on our findings in the stable A3H humanized mice infected with NLCSFV3 (Fig 3 & S6 Table). Our simulations well reproduced that the prevalence of hyper HIV-1 in the human population with different stable A3H proportion (Fig 5E).

### Statistics

The data are presented as averages ± SDs or SEMs. Statistically significant differences were determined by Student's *t* test, Paired *t* test, and Mann-Whitney U test. To determine statistically significant correlations (S9 Fig), the Spearman rank correlation test was applied to the data.

### Accession number

An accession number for the data generated in this study is as follows: the RNA-seq data of the splenic MNCs of HIV-1-infected (n = 4) and mock-infected (n = 4) humanized mice (GEO: GSE92262).

## Supporting information

S1 Fig Evaluation of anti-A3 activity of Vif derivatives detected in infected humanized mice. The expression plasmids of the Vif derivatives were cotransfected with pNLCSFV3Δ*vif* and either with or without expression plasmids for Flag-tagged A3D (50 ng), A3F (10 ng) or A3G (10 ng) into HEK293T cells. The infectivity of released virions was determined by using TZM-bl cells. *P < 0.05 versus "no A3/no Vif" by Student's *t* test. The assay was performed in triplicate. The data represents average with SD. NS, no statistic difference versus "no A3/no Vif". The symbols are identical to those in Fig 1E.
(TIF)

S2 Fig Real-time RT-PCR of *A3D*, *A3F* and *A3G*. (**A**) Splenic human CD4^+^ T cells (CD45^+^ CD3^+^ CD8^−^ cells) of mock-infected mice (n = 7) and HIV-1-infected mice (n = 13) were sorted using FACSJazz and the mRNA expression levels of *A3D*, *A3F* and *A3G* were analyzed by real-time RT-PCR as described in Materials and Methods. The value of mock-infected mice is set as 1. *P < 0.05 versus mock-infected mice by Mann-Whitney U test. (**B**) The mRNA expression levels of *A3D*, *A3F* and *A3G* in infected mice expressing intermediate A3H (n = 7) and stable A3H (n = 6) were analyzed by real-time RT-PCR. NS, no statistic difference. AU, arbitrary unit.
(TIF)

S3 Fig Sensitivity of the 3 IMCs to A3H-II. The IMCs (strains NLCSFV3, JRCSF, AD8, *vif-*deleted NLCSFV3, hyper NLCSFV3 and hypo NLCSFV3) were cotransfected either with Flag-tagged A3H-II expression plasmid at 4 different amounts (0, 25, 50, and 100 ng) into HEK293T cells. The assay was performed in triplicate. The infectivity of released virus was determined by using TZM-bl cells, and the percentage of the value of "no A3H-II" is shown. The data represents average with SD. The horizontal broken line represents 100%. Note that various versions of NLCSFV3 differ only in *vif*".
(TIF)

S4 Fig Viral *vif* sequences in the infected mice with stable A3H. The *vif* ORF of viral RNA in the plasma of infected mice (Fig 3C) were analyzed. Raw data (**A**) and mutation matrix (**B**) are respectively shown.
(TIF)

S5 Fig Viral *vif* sequences in the infected mice with intermediate A3H. (**A**) Phylogenetic trees of *vif* sequence. Viral *vif* sequences in the plasma of infected mice at 6 wpi were analyzed as described in Materials and Methods. Results of 3 infected mice with intermediate A3H (mice #50–52) are respectively shown. Scale bar represents one nucleotide substitution. Raw data (**B**) and mutation matrix (**C**) are also shown.
(TIF)

S6 Fig Up-regulation of *A3H* expression by activation stimuli. (**A, B**) Activation and up-regulation of *A3H* expression in *in vitro* human CD4^+^ T cell culture. (**A**) Human peripheral CD4^+^ T cells (n = 5) were stimulated with anti-CD3/anti-CD28 dynabeads as previously described [23], and the activation status was analyzed by staining with CD25. Representative dot plots of flow cytometry (left) and the summarized results (right) are shown. (**B**) The mRNA expression level of *A3H* in the human peripheral CD4^+^ T cells with or without stimulation of anti-CD3/anti-CD28 dynabeads (n = 5 each) was analyzed by real-time RT-PCR as described in Materials and Methods. The average value of non-stimulated CD4^+^ T cells is set as 1. Paired *t* test was applied to determine statistically significant difference. (**C**) Activation status of the human CD4^+^ T cells of humanized mice. Splenic human CD4^+^ T cells of humanized mice (n = 10) and the human peripheral CD4^+^ T cells with or without stimulation of anti-CD3/anti-CD28 dynabeads (n = 5 each) were stained with intracellular Ki67, an activation marker, and its expression level was analyzed by flow cytometry. Representative dot plots of flow cytometry (left) and the summarized results (right) are shown. In panels **A** and **C**, horizontal bars represent averages with SEMs. The numbers on each dot plot indicates the percentage of gated cells.
(TIF)

S7 Fig Representative of live cell sorting. Representative dot plots for cell sorting are shown. The numbers on each dot plot indicates the percentage of gated cells.
(TIF)

S8 Fig Conservation of the Vif responsible residues to counteract stable A3H. The Vif ORF sequences of HIV-1 group M (n = 2,976; one sequence per patient) were extracted from the database and aligned as described in Materials and Methods. The logoplot of Vif amino acid sequence is constructed using WebLogo 3 (http://weblogo.threeplusone.com) and the residues at positions 25–55 are shown. The two amino acids responsible for stable A3H counteraction (residues 39 and 48) are indicated in red. As a control, the YRHHY motif (residues 40–44) that is responsible for A3G counteraction is indicated in blue.
(TIF)

S9 Fig Correlation between the percentages of hyper HIV-1 and stable A3H individuals in the world. The percentage of hyper Vif (y-axis) and the proportion of stable A3H individuals (x-axis) in each region and country are respectively extracted from the database. To determine statistically significant correlations, the Spearman rank correlation test was applied to the data. See also S6 & S7 Tables.
(TIF)

S10 Fig No Vif reversion in the humanized mice infected with 4A HIV-1. Phylogenetic trees of *vif* sequence. Viral *vif* sequences in the plasma of humanized mice infected wit 4A HIV-1, which is incapable of counteracting A3F, at 6 wpi were analyzed as described in Materials and Methods. Results of 2 infected mice with intermediate A3H (mice #65 and #66) are respectively shown. The wild-type NLCSFV3 *vif* sequence was used as the outgroup. Scale bar represents one nucleotide substitution.
(TIF)

S1 Table Humanized mice used in Figs 1 & 2. A full list of the 14 humanized mice used in Figs 1 & 2.
(PDF)

S2 Table Percentage of hyper Vif derivatives in stable A3H humanized mice co-inoculated with hyper and hypo HIV-1s. A full list of the percentages of hyper Vif derivatives in the 8 stable A3H humanized mice (Fig 1).
(PDF)

S3 Table Summary of the sequences of hypo Vif derivatives detected in intermediate A3H humanized mice co-inoculated with hyper and hypo HIV-1s. A full list of hypo *vif* derivatives in the 6 intermediate A3H humanized mice.
(PDF)

S4 Table Percentage of hyper Vif derivatives in intermediate A3H humanized mice co-inoculated with hyper and hypo HIV-1s. A full list of the percentages of hyper Vif derivatives in the 6 intermediate A3H humanized mice (Fig 2).
(PDF)

S5 Table Humanized mice used in Fig 3 & S10 Fig. A full list of the 52 humanized mice used in Fig 3 & S10 Fig.
(PDF)

S6 Table Percentage of hyper Vif derivatives in stable A3H humanized mice infected with HIV-1 NLCSFV3. A full list of the percentages of hyper Vif derivatives in the 4 stable A3H humanized mice (Fig 3).
(PDF)

S7 Table Percentage of hyper Vif derivatives in intermediate A3H humanized mice infected with HIV-1 NLCSFV3. A full list of the percentages of hyper Vif derivatives in the 3 intermediate A3H humanized mice (S5 Fig).
(PDF)

S8 Table Top 50 annotations of GSEA analysis. A full list of the top 50 annotations of GSEA analysis.
(PDF)

S9 Table Amino acid residues positioned at 39 and 48 of the 24 IMCs used in this study. A full list of the amino acid residues positioned at 39 and 48 of the 24 IMCs used in this study.
(PDF)

S10 Table Percentage of hyper Vif in HIV-1 subtypes and groups deposited in HIV-1 sequence database. A full list of the percentages of hyper Vif in HIV-1 subtypes and groups deposited in Los Alamos National Laboratory HIV-1 sequence database (https://www.hiv.lanl.gov/components/sequence/HIV/search/search.html).
(PDF)

S11 Table Proportion of stable A3H individuals in each population and region. A full list of the proportion of stable A3H individuals in each population and region from 1000 Genomes Project (http://www.internationalgenome.org).
(PDF)

S12 Table Proportions of hyper HIV-1 and stable A3H individuals in each region and country. A full list of the proportions of hyper HIV-1 and stable A3H individuals in each region and country.
(PDF)
