# Correction: HIV-1 competition experiments in humanized mice show that APOBEC3H imposes selective pressure and promotes virus adaptation

**DOI:** 10.1371/journal.ppat.1006606

**Published:** 2017-09-07

**Authors:** Yusuke Nakano, Naoko Misawa, Guillermo Juarez-Fernandez, Miyu Moriwaki, Shinji Nakaoka, Takaaki Funo, Eri Yamada, Andrew Soper, Rokusuke Yoshikawa, Diako Ebrahimi, Yuuya Tachiki, Shingo Iwami, Reuben S. Harris, Yoshio Koyanagi, Kei Sato

The following information is missing from the Funding section: This study was supported by the UCLA CFAR Gene and Cellular Therapy Core Facility (UCLA Center for AIDS Research NIH/NIAID 5P30 AI028697).
