# Supplementary figures and images for: HIV-1 competition experiments in humanized mice show that APOBEC3H imposes selective pressure and promotes virus adaptation
Source: PLoS Pathog. 2017 May 5;13(5):e1006348. doi: 10.1371/journal.ppat.1006348 (PMC5435363; doi:10.1371/journal.ppat.1006348)

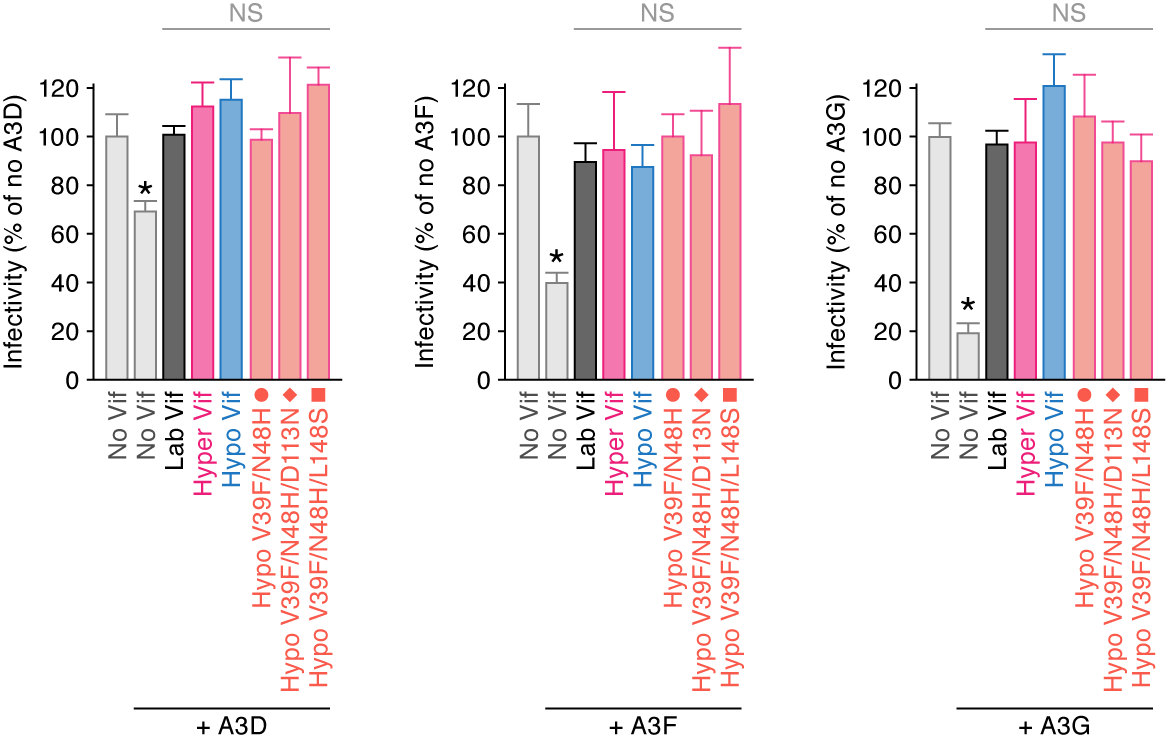

Supplement: S1 Fig — The expression plasmids of the Vif derivatives were cotransfected with pNLCSFV3Δvif and either with or without expression plasmids for Flag-tagged A3D (50 ng), A3F (10 ng) or A3G (10 ng) into HEK293T cells. The infectivity of released virions was determined by using TZM-bl cells. *P < 0.05 versus "no A3/no Vif" by Student's t test. The assay was performed in triplicate. The data represents average with SD. NS, no statistic difference versus "no A3/no Vif". The symbols are identical to those in Fig 1E. (TIF) [file ppat.1006348.s001.tif]

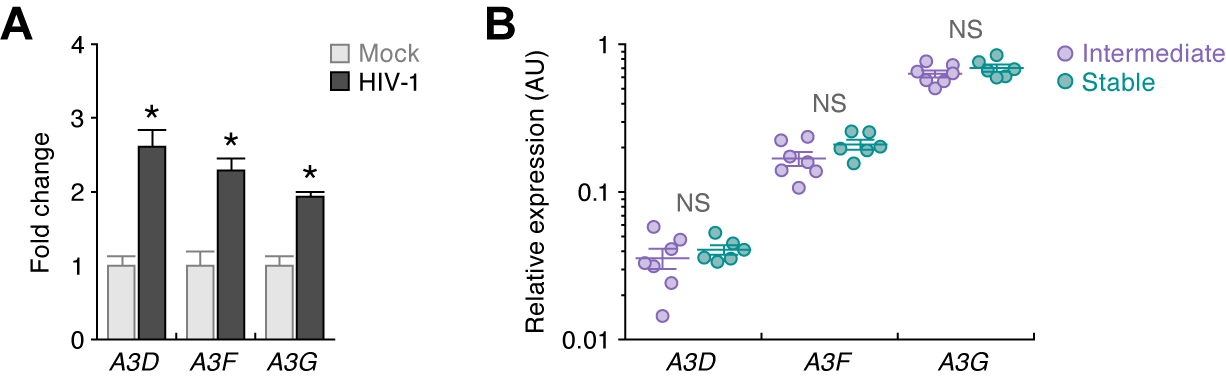

Supplement: S2 Fig — (A) Splenic human CD4+ T cells (CD45+ CD3+ CD8− cells) of mock-infected mice (n = 7) and HIV-1-infected mice (n = 13) were sorted using FACSJazz and the mRNA expression levels of A3D, A3F and A3G were analyzed by real-time RT-PCR as described in Materials and Methods. The value of mock-infected mice is set as 1. *P < 0.05 versus mock-infected mice by Mann-Whitney U test. (B) The mRNA expression levels of A3D, A3F and A3G in infected mice expressing intermediate A3H (n = 7) and stable A3H (n = 6) were analyzed by real-time RT-PCR. NS, no statistic difference. AU, arbitrary unit. (TIF) [file ppat.1006348.s002.tif]

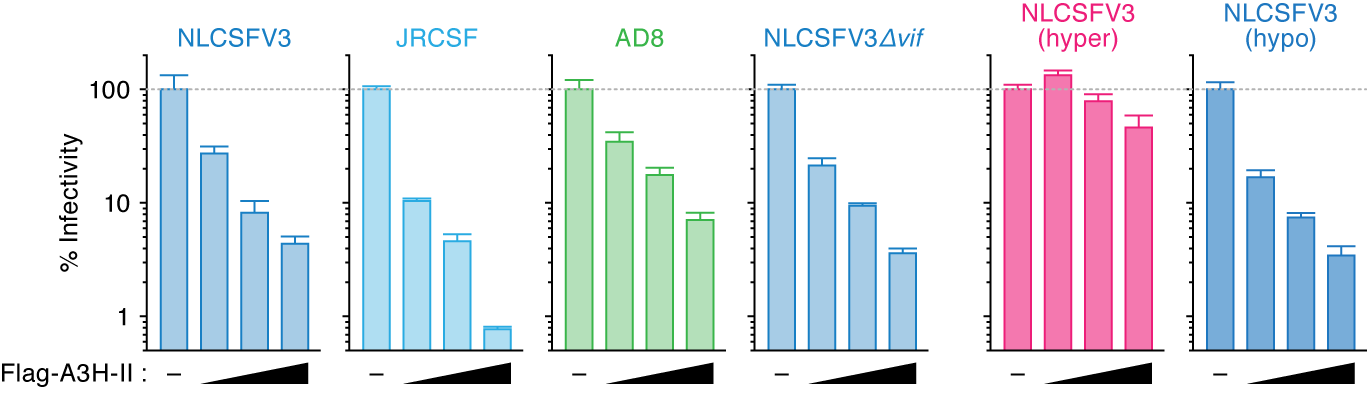

Supplement: S3 Fig — The IMCs (strains NLCSFV3, JRCSF, AD8, vif-deleted NLCSFV3, hyper NLCSFV3 and hypo NLCSFV3) were cotransfected either with Flag-tagged A3H-II expression plasmid at 4 different amounts (0, 25, 50, and 100 ng) into HEK293T cells. The assay was performed in triplicate. The infectivity of released virus was determined by using TZM-bl cells, and the percentage of the value of "no A3H-II" is shown. The data represents average with SD. The horizontal broken line represents 100%. Note that various versions of NLCSFV3 differ only in vif". (TIF) [file ppat.1006348.s003.tif]

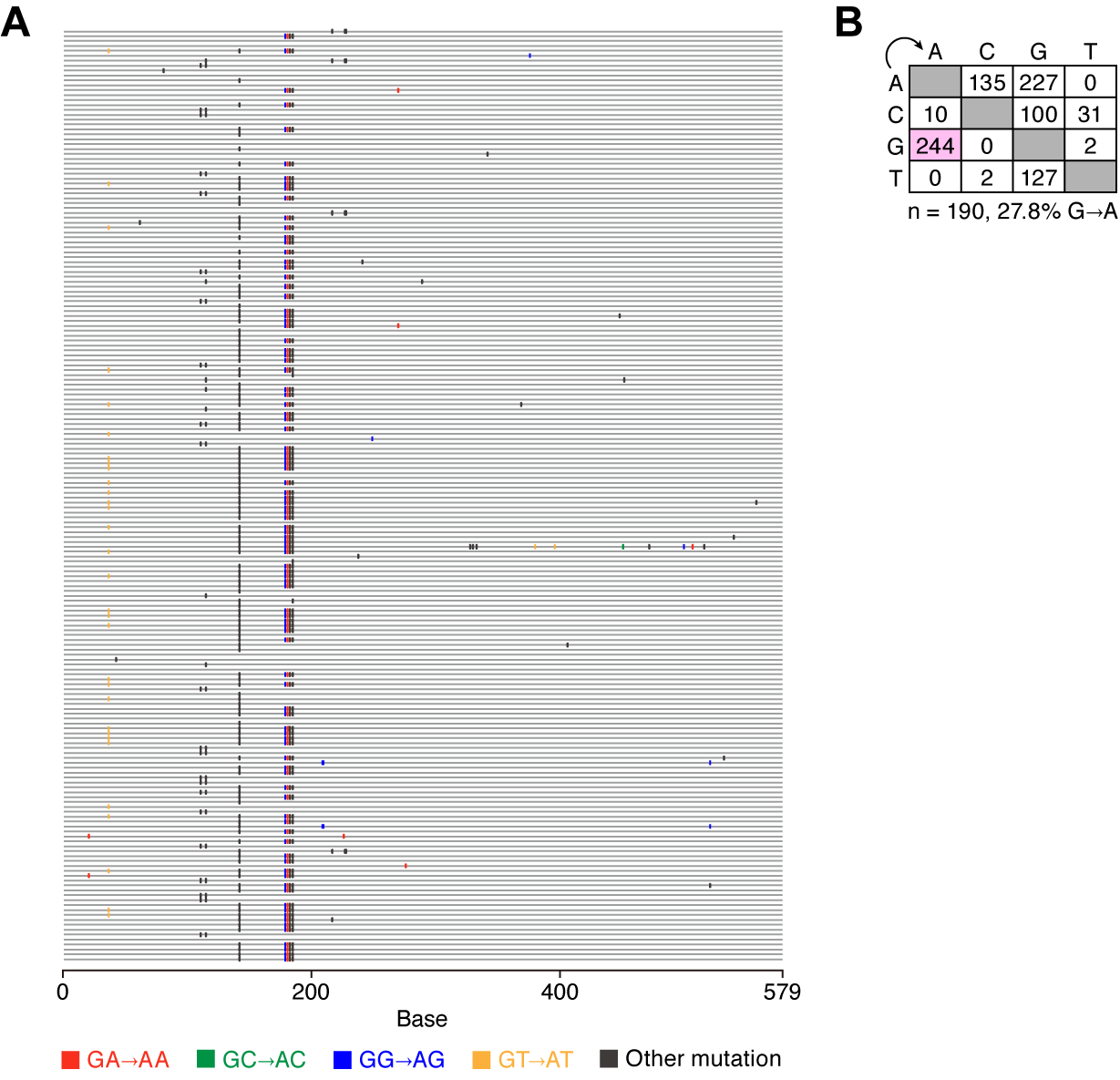

Supplement: S4 Fig — The vif ORF of viral RNA in the plasma of infected mice (Fig 3C) were analyzed. Raw data (A) and mutation matrix (B) are respectively shown. (TIF) [file ppat.1006348.s004.tif]

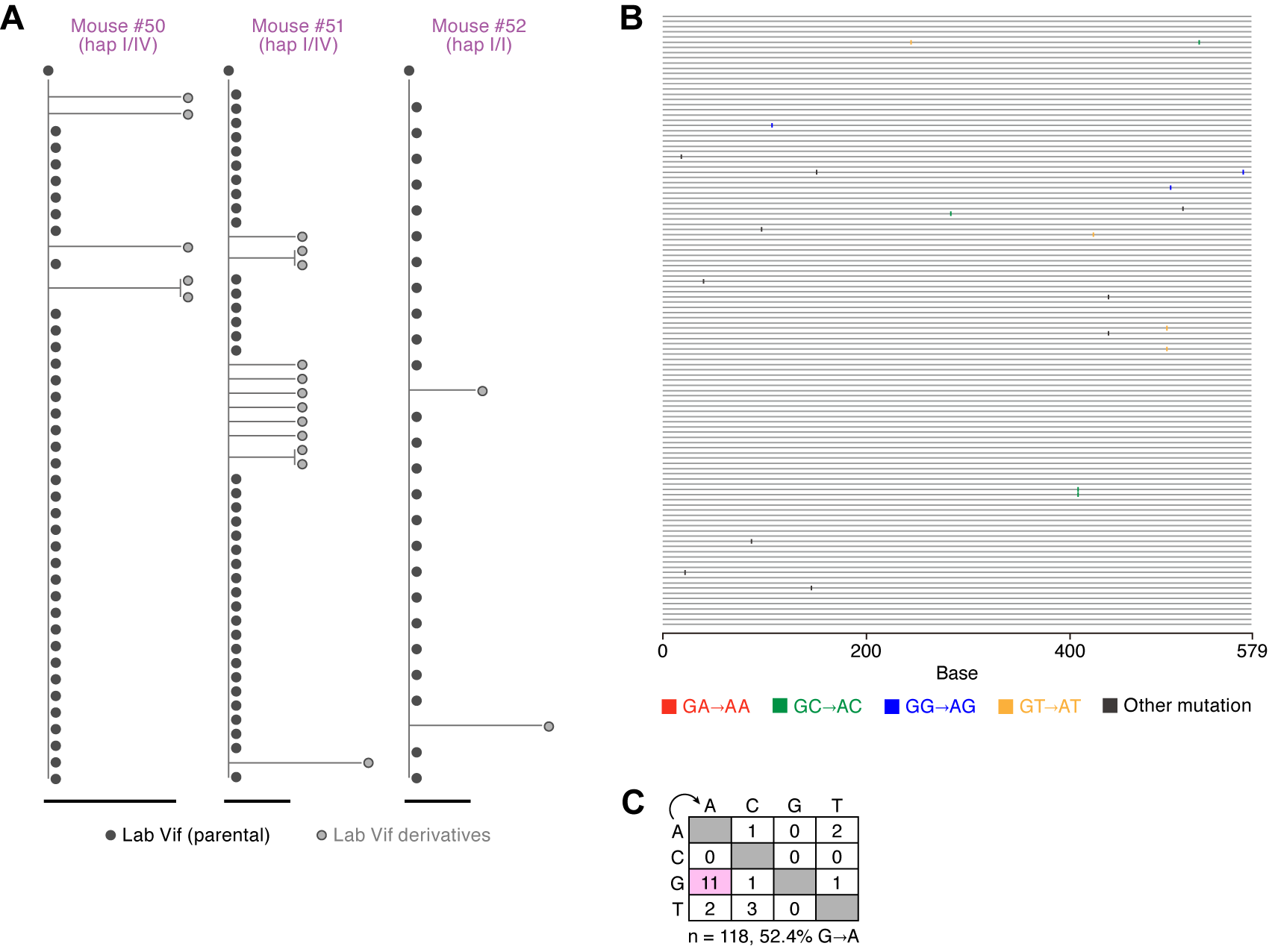

Supplement: S5 Fig — (A) Phylogenetic trees of vif sequence. Viral vif sequences in the plasma of infected mice at 6 wpi were analyzed as described in Materials and Methods. Results of 3 infected mice with intermediate A3H (mice #50–52) are respectively shown. Scale bar represents one nucleotide substitution. Raw data (B) and mutation matrix (C) are also shown. (TIF) [file ppat.1006348.s005.tif]

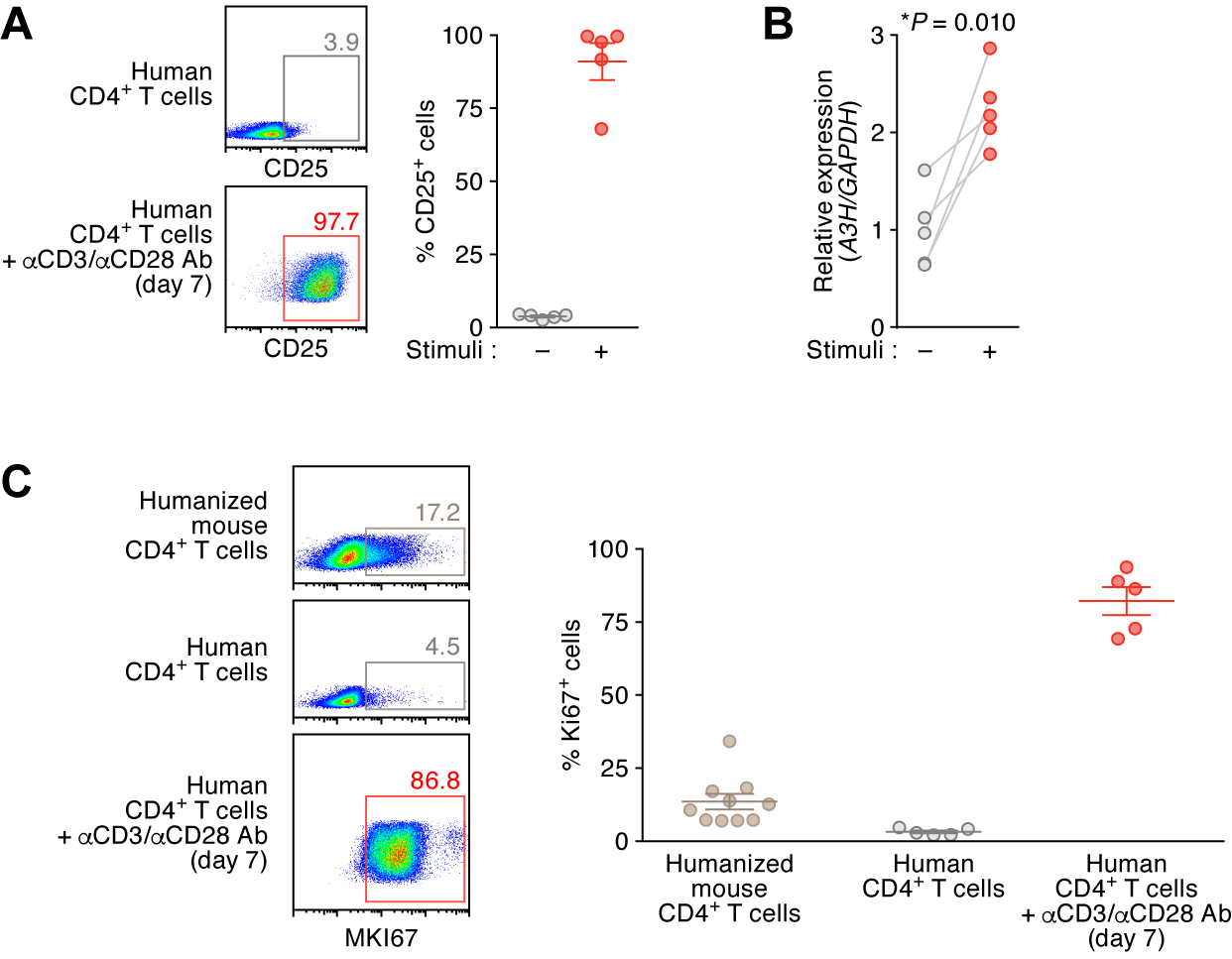

Supplement: S6 Fig — (A, B) Activation and up-regulation of A3H expression in in vitro human CD4+ T cell culture. (A) Human peripheral CD4+ T cells (n = 5) were stimulated with anti-CD3/anti-CD28 dynabeads as previously described [23], and the activation status was analyzed by staining with CD25. Representative dot plots of flow cytometry (left) and the summarized results (right) are shown. (B) The mRNA expression level of A3H in the human peripheral CD4+ T cells with or without stimulation of anti-CD3/anti-CD28 dynabeads (n = 5 each) was analyzed by real-time RT-PCR as described in Materials and Methods. The average value of non-stimulated CD4+ T cells is set as 1. Paired t test was applied to determine statistically significant difference. (C) Activation status of the human CD4+ T cells of humanized mice. Splenic human CD4+ T cells of humanized mice (n = 10) and the human peripheral CD4+ T cells with or without stimulation of anti-CD3/anti-CD28 dynabeads (n = 5 each) were stained with intracellular Ki67, an activation marker, and its expression level was analyzed by flow cytometry. Representative dot plots of flow cytometry (left) and the summarized results (right) are shown. In panels A and C, horizontal bars represent averages with SEMs. The numbers on each dot plot indicates the percentage of gated cells. (TIF) [file ppat.1006348.s006.tif]

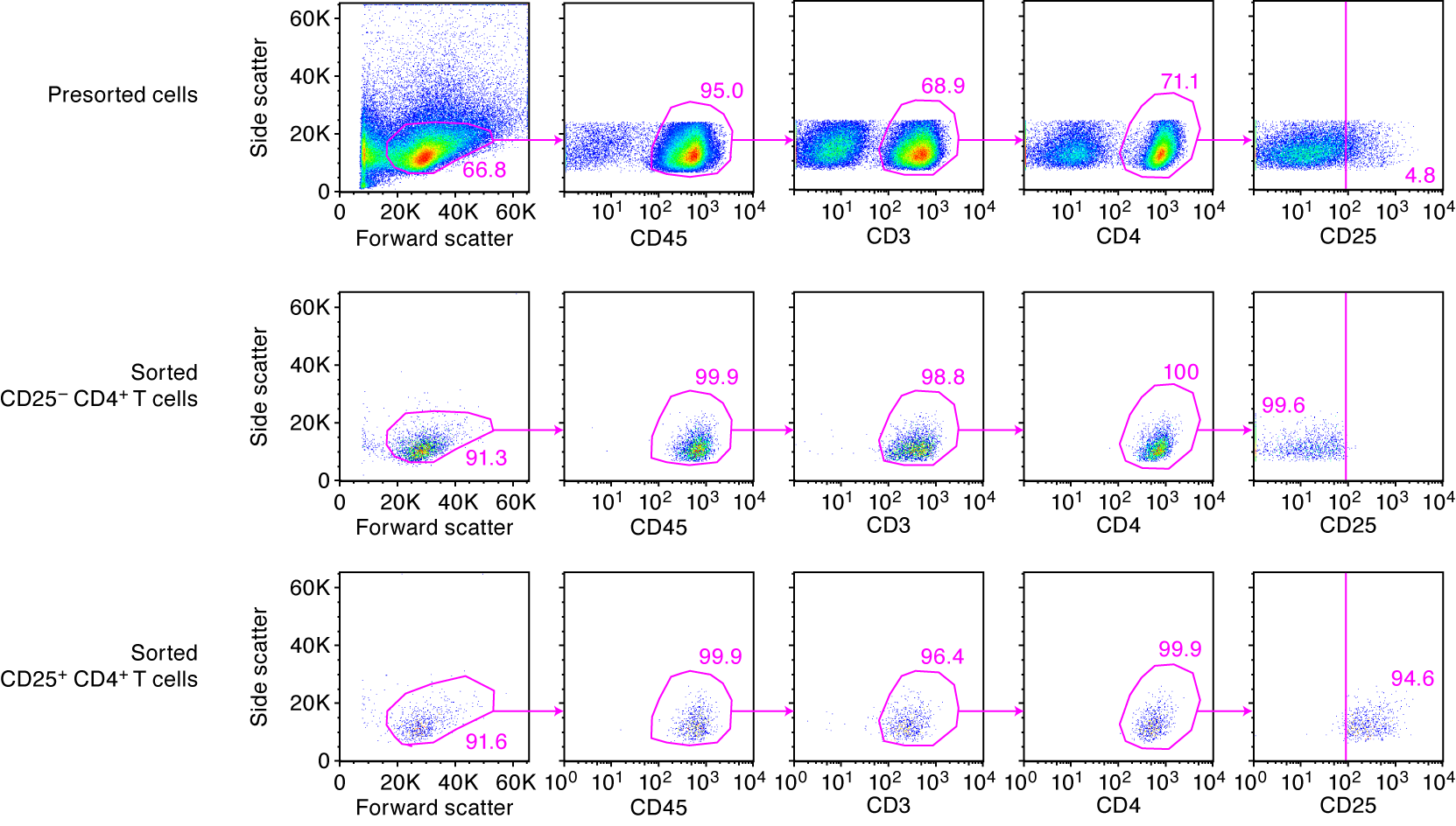

Supplement: S7 Fig — Representative dot plots for cell sorting are shown. The numbers on each dot plot indicates the percentage of gated cells. (TIF) [file ppat.1006348.s007.tif]

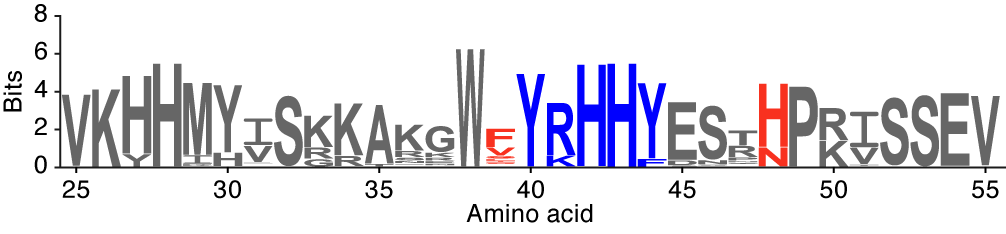

Supplement: S8 Fig — The Vif ORF sequences of HIV-1 group M (n = 2,976; one sequence per patient) were extracted from the database and aligned as described in Materials and Methods. The logoplot of Vif amino acid sequence is constructed using WebLogo 3 (http://weblogo.threeplusone.com) and the residues at positions 25–55 are shown. The two amino acids responsible for stable A3H counteraction (residues 39 and 48) are indicated in red. As a control, the YRHHY motif (residues 40–44) that is responsible for A3G counteraction is indicated in blue. (TIF) [file ppat.1006348.s008.tif]

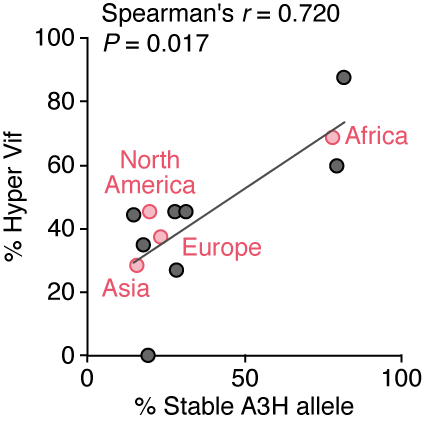

Supplement: S9 Fig — The percentage of hyper Vif (y-axis) and the proportion of stable A3H individuals (x-axis) in each region and country are respectively extracted from the database. To determine statistically significant correlations, the Spearman rank correlation test was applied to the data. See also S6 & S7 Tables. (TIF) [file ppat.1006348.s009.tif]

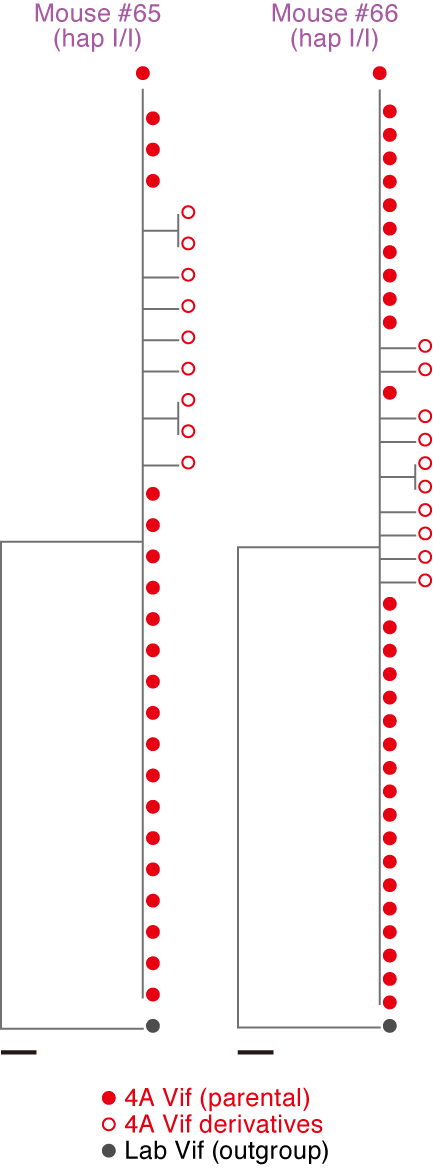

Supplement: S10 Fig — Phylogenetic trees of vif sequence. Viral vif sequences in the plasma of humanized mice infected wit 4A HIV-1, which is incapable of counteracting A3F, at 6 wpi were analyzed as described in Materials and Methods. Results of 2 infected mice with intermediate A3H (mice #65 and #66) are respectively shown. The wild-type NLCSFV3 vif sequence was used as the outgroup. Scale bar represents one nucleotide substitution. (TIF) [file ppat.1006348.s010.tif]
