# Supplementary material for: HIV-1 competition experiments in humanized mice show that APOBEC3H imposes selective pressure and promotes virus adaptation
Source: PLoS Pathog. 2017 May 5;13(5):e1006348. doi: 10.1371/journal.ppat.1006348 (PMC5435363; doi:10.1371/journal.ppat.1006348)
Supplement: S1 Table — A full list of the 14 humanized mice used in Figs 1 & 2. (PDF) [file ppat.1006348.s011.pdf]

Table S1. Humanized mice used in Figures 1 and 2.

Mouse no.	Recipient mouse		Transplanted hHSCs§		*APOBEC3H* haplotype		Inoculated age (weeks)
	Lot no.*	Sex†	Donor lot‡	No. of cells			
1	235	F	A	150,000	III / **V**	Stable	18
2	235	M	A	150,000	III / **V**	Stable	18
3	238	F	B	120,000	I / **II**	Stable	13
4	238	F	B	120,000	I / **II**	Stable	13
5	238	M	B	120,000	I / **II**	Stable	13
6	243	M	B	230,000	I / **II**	Stable	14
7	243	M	B	230,000	I / **II**	Stable	14
8	243	M	B	230,000	I / **II**	Stable	14
9	234	M	C	135,000	I / I	Intermediate	18
10	253	M	C	160,000	I / I	Intermediate	15
11	253	M	C	160,000	I / I	Intermediate	15
12	253	M	C	160,000	I / I	Intermediate	15
13	241	F	D	140,000	I / VI	Intermediate	14
14	250	M	E	100,000	I / I	Intermediate	14

* Seven lots of newborn NOG mice were used for the recipient.
† F, female; M, male.
‡ NOG-hCD34 mice were reconstructed with one of 5 donors.
§ hHSCs, human CD34$^+$ hematopoietic stem cells.
