# Supplementary material for: HIV-1 competition experiments in humanized mice show that APOBEC3H imposes selective pressure and promotes virus adaptation
Source: PLoS Pathog. 2017 May 5;13(5):e1006348. doi: 10.1371/journal.ppat.1006348 (PMC5435363; doi:10.1371/journal.ppat.1006348)
Supplement: S2 Table — A full list of the percentages of hyper Vif derivatives in the 8 stable A3H humanized mice (Fig 1). (PDF) [file ppat.1006348.s012.pdf]

Table S2. Percentage of hyper Vif derivatives in stable A3H humanized mice co-inoculated with hyper and hypo HIV-1s.

Mouse no.*	Donor lot*	*APOBE3H* haplotype		# Analyzed sequence	# Hyper Vif derivatives	% Hyper Vif derivatives	#Hypo Vif derivatives	% Hypo Vif derivatives	# Hypo Vif derivatives with anti-A3H activity†	**% Vif with anti-A3H-II activity‡**
1	A	III / **V**	Stable	38	35	92.1	3	7.9	1	**94.7**
2	A	III / **V**	Stable	64	40	62.5	24	37.5	1	**64.1**
3	B	I / **II**	Stable	52	30	57.7	22	42.3	1	**59.6**
4	B	I / **II**	Stable	49	43	87.8	6	12.2	0	**87.8**
5	B	I / **II**	Stable	52	17	32.7	35	67.3	23	**76.9**
6	B	I / **II**	Stable	47	44	93.6	3	6.4	0	**93.6**
7	B	I / **II**	Stable	40	34	85.0	6	15.0	2	**90.0**
8	B	I / **II**	Stable	49	43	87.8	6	12.2	0	**87.8**
			**Average**	391	286	**73.1**	105	**26.9**	28	**80.3**
			**SEM**			**7.7**		**7.7**		**4.8**

* Identical to Figure 1 and Table S1.

† Number of hyper Vif derivatives acquiring anti-A3H-II activity (Figures 1E-1G).

‡ Percentage of hyper Vif derivatives and the hyper Vif derivatives acquiring anti-A3H-II activity.
