# Supplementary material for: HIV-1 competition experiments in humanized mice show that APOBEC3H imposes selective pressure and promotes virus adaptation
Source: PLoS Pathog. 2017 May 5;13(5):e1006348. doi: 10.1371/journal.ppat.1006348 (PMC5435363; doi:10.1371/journal.ppat.1006348)
Supplement: S3 Table — A full list of hypo vif derivatives in the 6 intermediate A3H humanized mice. (PDF) [file ppat.1006348.s013.pdf]

Table S3. Sumary of the sequences of hypo Vif derivatives detected in intermediate A3H humanized mice co-inoculated with hyper and hypo HIV-1s.

Mouse no.	Sequence (nucleotide)	# detected
9	Parental	19
	g11a	1
	g21a/a96g/g115t/g226a	1
	t88c	2
	g115t	1
	a142c/g480a	1
	c316t	1
	t321c/g480a	1
	c356t	1
	a401g	1
	a503t	1
	a565g	1
10	Parental	22
	t86c	1
	t92c/g115t/a142c	1
	g115t	1
	a275g	1
	a542t	1
	g551a	1
	g553a	1
11	Parental	22
	g115t	1
	c140a	4
	a142c	1
	a142c/c185g	1
	a302g	1
	t387c	3
	c406t	1
	a464c	1
12	Parental	21
	c129a/c140t/a263g	1
	a142c	4
	g181a	3
	c214a	1
	a263g	2
	t339c	2
13	Parental	13
	t29c	1
	t93c	1
	c185g	2

Note that hypo Vif derivatives were not detected in mouse no. 14.
