# Supplementary material for: HIV-1 competition experiments in humanized mice show that APOBEC3H imposes selective pressure and promotes virus adaptation
Source: PLoS Pathog. 2017 May 5;13(5):e1006348. doi: 10.1371/journal.ppat.1006348 (PMC5435363; doi:10.1371/journal.ppat.1006348)
Supplement: S4 Table — A full list of the percentages of hyper Vif derivatives in the 6 intermediate A3H humanized mice (Fig 2). (PDF) [file ppat.1006348.s014.pdf]

Table S4. Percentage of hyper Vif derivatives in intermediate A3H humanized mice co-inoculated with hyper and hypo HIV-1s.

Mouse no.*	Donor lot*	*APOBE3H* haplotype		# Analyzed sequence	# Hyper Vif derivatives	% Hyper Vif derivatives	#Hypo Vif derivatives	% Hypo Vif derivatives	# Hypo Vif derivatives with anti-A3H activity	**% Vif with anti-A3H-II activity†**
9	C	I / I	Intermediate	33	2	6.1	31	93.9	0	**6.1**
10	C	I / I	Intermediate	47	18	38.3	29	61.7	0	**38.3**
11	C	I / I	Intermediate	49	14	28.6	35	71.4	0	**28.6**
12	C	I / I	Intermediate	47	13	27.7	34	72.3	0	**27.7**
13	D	I / VI	Intermediate	50	33	66.0	17	34.0	0	**66.0**
14	E	I / I	Intermediate	47	47	100.0	0	0.0	0	**100.0**
			**Average**	273	127	**46.5**	146	**53.5**		**46.5**
			**SEM**			**13.7**		**13.7**		**13.7**

* Identical to Figure 2 and Table S1.

† Percentage of hyper Vif derivatives and the hypo Vif derivatives acquiring anti-A3H-II activity.
