# Supplementary material for: HIV-1 competition experiments in humanized mice show that APOBEC3H imposes selective pressure and promotes virus adaptation
Source: PLoS Pathog. 2017 May 5;13(5):e1006348. doi: 10.1371/journal.ppat.1006348 (PMC5435363; doi:10.1371/journal.ppat.1006348)
Supplement: S5 Table — A full list of the 52 humanized mice used in Fig 3 & S10 Fig. (PDF) [file ppat.1006348.s015.pdf]

Table S5. Humanized mice used in Figures 3 and S10.

Mouse no.	Recipient mouse		Transplanted hHSCs§ (donor lot‡)	*APOBEC3H* haplotype		Peak VL#	Virus inoculated	Reference
	Lot no.*	Sex†						
15	105	M	F	**II** / **II**	Stable	5.90	AD8	23
16	105	M	F	**II** / **II**	Stable	6.68	AD8	23
17	128	F	G	**II** / IV	Stable	6.59	AD8	23
18	128	M	G	**II** / IV	Stable	6.34	AD8	23
19	129	F	G	**II** / IV	Stable	6.75	AD8	23
20	129	M	G	**II** / IV	Stable	6.01	AD8	23
21	80	M	H	I / **II**	Stable	5.56	JRCSF	12
22	156	F	H	I / **II**	Stable	4.72	NLCSFV3	24
23	156	M	H	I / **II**	Stable	5.20	NLCSFV3	24
24	157	F	H	I / **II**	Stable	5.39	NLCSFV3	24
25	157	M	H	I / **II**	Stable	5.60	NLCSFV3	24
26	160	F	H	I / **II**	Stable	4.81	NL4-3	12
27	160	M	H	I / **II**	Stable	4.78	NL4-3	12
28	108	F	I	I / I	Intermediate	5.75	AD8	23
29	108	M	I	I / I	Intermediate	5.27	AD8	23
30	119	F	I	I / I	Intermediate	6.06	AD8	23
31	106	M	J	I / IV	Intermediate	5.76	AD8	23
32	110	M	K	I / IV	Intermediate	4.58	AD8	23
33	110	M	K	I / IV	Intermediate	4.64	AD8	23
34	126	M	L	I / I	Intermediate	6.68	AD8	23
35	126	M	L	I / I	Intermediate	4.40	AD8	23
36	126	F	L	I / I	Intermediate	5.60	AD8	23
37	127	F	L	I / I	Intermediate	5.86	AD8	23
38	127	F	L	I / I	Intermediate	6.41	AD8	23
39	168	F	M	I / III	Intermediate	6.33	AD8	This study
40	168	F	M	I / III	Intermediate	6.68	AD8	This study
41	173	F	M	I / III	Intermediate	5.37	AD8	This study
42	191	M	N	I / I	Intermediate	6.55	AD8	This study
43	192	M	N	I / I	Intermediate	6.03	AD8	This study
44	192	M	N	I / I	Intermediate	5.86	AD8	This study
45	165	M	M	I / III	Intermediate	6.47	JRCSF	This study
46	179	M	O	I / IV	Intermediate	6.51	JRCSF	This study
47	191	F	P	I / I	Intermediate	6.19	JRCSF	This study
48	191	F	P	I / I	Intermediate	6.04	JRCSF	This study
49	191	M	P	I / I	Intermediate	6.51	JRCSF	This study
50	136	F	Q	I / IV	Intermediate	5.95	NLCSFV3	24
51	136	M	Q	I / IV	Intermediate	6.37	NLCSFV3	24
52	135	F	L	I / I	Intermediate	5.81	NLCSFV3	24
53	224	M	R	I / I	Intermediate	6.72	NLCSFV3	This study
54	225	F	S	I / I	Intermediate	5.88	NLCSFV3	This study
55	227	F	T	I / IV	Intermediate	5.11	NLCSFV3	This study
56	159	F	M	I / III	Intermediate	3.96	NL4-3	12
57	159	F	M	I / III	Intermediate	5.68	NL4-3	12
58	165	M	M	I / III	Intermediate	4.96	NL4-3	12
59	162	F	U	I / IV	Intermediate	6.13	NL4-3	12
60	162	M	U	I / IV	Intermediate	5.47	NL4-3	12
61	163	F	U	I / IV	Intermediate	5.56	NL4-3	12
62	163	F	U	I / IV	Intermediate	4.56	NL4-3	12
63	164	F	U	I / IV	Intermediate	5.75	NL4-3	12
64	166	F	U	I / IV	Intermediate	5.83	NL4-3	12
65	135	M	L	I / I	Intermediate	6.11	4A	24
66	225	F	T	I / IV	Intermediate	6.12	4A	This study

* Twenty-nine lots of newborn NOG mice were used for the recipient.

† F, female; M, male.

‡ NOG-hCD34 mice were reconstructed with one of 16 donors.

§ hHSCs, human $CD34^+$ hematopoietic stem cells.

# Peal viral load, copies/ml plasma (log10).
