# Supplementary material for: HIV-1 competition experiments in humanized mice show that APOBEC3H imposes selective pressure and promotes virus adaptation
Source: PLoS Pathog. 2017 May 5;13(5):e1006348. doi: 10.1371/journal.ppat.1006348 (PMC5435363; doi:10.1371/journal.ppat.1006348)
Supplement: S6 Table — A full list of the percentages of hyper Vif derivatives in the 4 stable A3H humanized mice (Fig 3). (PDF) [file ppat.1006348.s016.pdf]

Table S6. Percentage of hyper Vif derivatives in stable A3H humanized mice infected with HIV-1 NLCSFV3.

Mouse no.*	Donor lot*	*APOBE3H* haplotype		# Analyzed sequence	# Vif derivatives with anti-A3H activity†	**% Vif with anti-A3H-II activity‡**
22	H	I / **II**	Stable	58	32	**55.2**
23	H	I / **II**	Stable	60	39	**65.0**
24	H	I / **II**	Stable	64	45	**70.3**
25	H	I / **II**	Stable	61	29	**47.5**
			**Average**	243	145	**59.7**
			**SEM**			**5.1**

* Identical to Figure 3 and Table S5.

† Number of Vif derivatives acquiring anti-A3H-II activity (Figures 3C-3E).

‡ Percentage of Vif derivatives acquiring anti-A3H-II activity.
