# Supplementary material for: HIV-1 competition experiments in humanized mice show that APOBEC3H imposes selective pressure and promotes virus adaptation
Source: PLoS Pathog. 2017 May 5;13(5):e1006348. doi: 10.1371/journal.ppat.1006348 (PMC5435363; doi:10.1371/journal.ppat.1006348)
Supplement: S7 Table — A full list of the percentages of hyper Vif derivatives in the 3 intermediate A3H humanized mice (S5 Fig). (PDF) [file ppat.1006348.s017.pdf]

Table S7. Percentage of hyper Vif derivatives in intermediate A3H humanized mice infected with HIV-1 NLCSFV3.

Mouse no.*	Donor lot*	*APOBE3H* haplotype		# Analyzed sequence	# Vif derivatives with anti-A3H activity	**% Vif with anti-A3H-II activity†**
50	Q	I / IV	Intermediate	42	0	**0.0**
51	Q	I / IV	Intermediate	49	0	**0.0**
52	L	I / I	Intermediate	27	0	**0.0**
			**Average**	118	0	**0.0**
			**SEM**			**0.0**

* Identical to Figure S5A and Table S5.

† Percentage of hyper Vif derivatives and the hyper Vif derivatives acquiring anti-A3H-II activity.
