# Supplementary material for: HIV-1 competition experiments in humanized mice show that APOBEC3H imposes selective pressure and promotes virus adaptation
Source: PLoS Pathog. 2017 May 5;13(5):e1006348. doi: 10.1371/journal.ppat.1006348 (PMC5435363; doi:10.1371/journal.ppat.1006348)
Supplement: S8 Table — A full list of the top 50 annotations of GSEA analysis. (PDF) [file ppat.1006348.s018.pdf]

Table S8. Top 50 annotations of GSEA analysis.

ID	Description*	Q value
**GO:0007159**	**leukocyte cell-cell adhesion**	**0.000029**
**GO:0050900**	**leukocyte migration**	**0.000199**
GO:0007229	integrin-mediated signaling pathway	0.000199
GO:0051270	regulation of cellular component movement	0.000199
GO:0002274	myeloid leukocyte activation	0.000266
GO:0040012	regulation of locomotion	0.000266
GO:0030334	regulation of cell migration	0.000270
GO:0050865	regulation of cell activation	0.000270
GO:2000145	regulation of cell motility	0.000366
GO:0002521	leukocyte differentiation	0.000405
GO:0002694	regulation of leukocyte activation	0.000470
GO:0050867	positive regulation of cell activation	0.000594
GO:0016337	cell-cell adhesion	0.001030
GO:0002696	positive regulation of leukocyte activation	0.001135
**GO:0042110**	**T cell activation**	**0.001651**
GO:0051249	regulation of lymphocyte activation	0.002116
**GO:0046649**	**lymphocyte activation**	**0.002116**
GO:0002252	immune effector process	0.002218
**GO:0006954**	**inflammatory response**	**0.002663**
**GO:0050852**	**T cell receptor signaling pathway**	**0.003578**
**GO:0030098**	**lymphocyte differentiation**	**0.003905**
GO:0051251	positive regulation of lymphocyte activation	0.003905
GO:0009617	response to bacterium	0.004720
GO:0007160	cell-matrix adhesion	0.005231
GO:0016044	cellular membrane organization	0.005231
GO:0061024	membrane organization	0.005343
GO:0042119	neutrophil activation	0.005619
GO:0002444	myeloid leukocyte mediated immunity	0.006113
GO:0007163	establishment or maintenance of cell polarity	0.006113
GO:0002757	immune response-activating signal transduction	0.006335
GO:0002764	immune response-regulating signaling pathway	0.006398
GO:0050870	positive regulation of T cell activation	0.006468
GO:0030099	myeloid cell differentiation	0.006607
GO:0051607	defense response to virus	0.007301
GO:0046631	alpha-beta T cell activation	0.007504
GO:0051272	positive regulation of cellular component movement	0.007651
GO:0031347	regulation of defense response	0.007942
GO:0002573	myeloid leukocyte differentiation	0.008997
GO:0001816	cytokine production	0.009114
GO:0050863	regulation of T cell activation	0.010051
GO:0030155	regulation of cell adhesion	0.010051
GO:0032675	regulation of interleukin-6 production	0.012167
**GO:0042102**	**positive regulation of T cell proliferation**	**0.013117**
**GO:0032635**	**interleukin-6 production**	**0.013117**
GO:0070661	leukocyte proliferation	0.013907
GO:0030029	actin filament-based process	0.013908
GO:0048872	homeostasis of number of cells	0.014327
GO:0030217	T cell differentiation	0.014680
GO:0002275	myeloid cell activation involved in immune response	0.017955
GO:0030168	platelet activation	0.017955

* The descriptions written in bold are summarized in Figure 4B.
