# Supplementary material for: HIV-1 competition experiments in humanized mice show that APOBEC3H imposes selective pressure and promotes virus adaptation
Source: PLoS Pathog. 2017 May 5;13(5):e1006348. doi: 10.1371/journal.ppat.1006348 (PMC5435363; doi:10.1371/journal.ppat.1006348)
Supplement: S9 Table — A full list of the amino acid residues positioned at 39 and 48 of the 24 IMCs used in this study. (PDF) [file ppat.1006348.s019.pdf]

Table S9. Amino acid residues positioned at 39 and 48 of the 24 IMCs used in this study.

Group	Subtype	Category*	Strain†	Residues		Anti-A3H-II activity‡	Reference§
				39	48		
M	B		Hyper HIV-1	F	H	+	25411794
M	B		Hypo HIV-1	V	N	–	25411794
M	B		NLCSFV3	F	N	–	9847335
M	B		JRCSF	I	N	–	3646751
M	B		AD8	V	N	–	8835195
M	B	TF	AD17	S	H	–	18256145
M	B	TF	CH470	F	H	+	23542380
M	B	TF	REJO	F	H	+	19487424
M	B	TF	RHPA	W	H	–	19487424
M	B	TF	CH058	F	H	+	19487424
M	B	TF	WITO	F	H	+	19487424
M	B	CC	CH058-6mo	F	H	+	19487424
M	B	CC	MCST	S	H	–	23542380
M	C	TF	ZM247v2	F	H	+	20485520
M	C	TF	CH042	F	H	+	23542380
M	C	TF	CH185	Y	H	+	23542380
M	C	TF	CH200v1	F	H	+	23542380
M	C	CC	CH269	F	H	+	23542380
M	C	CC	CH293	F	H	+	23542380
M	C	CC	CH440	F	H	+	23542380
N			DJO0131	Y	H	–	23308067
O			BCF183	Y	N	+	X
O			RBF206	L	N	+	X
P			RBF168	F	H	+	GQ328744

* TF, transmitted/founder; CC, chronic control.

† Hyper HIV-1 and hypo HIV-1 is based on strain NLCSFV3.

‡ Determined by the experiments shown in Figures 5C and S3.

§ For the referece of each virus, PMIDs (https://www.ncbi.nlm.nih.gov/pubmed) are listed. For RBF168, GenBank accession number is listed. X, not available.
