# Supplementary material for: HIV-1 competition experiments in humanized mice show that APOBEC3H imposes selective pressure and promotes virus adaptation
Source: PLoS Pathog. 2017 May 5;13(5):e1006348. doi: 10.1371/journal.ppat.1006348 (PMC5435363; doi:10.1371/journal.ppat.1006348)
Supplement: S10 Table — A full list of the percentages of hyper Vif in HIV-1 subtypes and groups deposited in Los Alamos National Laboratory HIV-1 sequence database (https://www.hiv.lanl.gov/components/sequence/HIV/search/search.html). (PDF) [file ppat.1006348.s020.pdf]

Table S10. Percentage of hyper Vif in HIV-1 subtypes and groups deposited in HIV-1 sequence database.

Group	Subtype	# Vif*		% Hyper Vif
		# Total	# Hyper Vif	
M	A1	199	96	**48.2**
M	A2	6	3	**50.0**
M	B	1954	739	**37.8**
M	C	566	434	**76.7**
M	D	74	17	**23.0**
M	F1	118	51	**43.2**
M	F2	8	8	**100**
M	G	42	28	**66.7**
M	H	5	3	**60.0**
M	J	2	0	**0**
M	K	2	1	**50.0**
M		2976	1380	**46.4**
N		7	6	**85.7**
O		51	0	**0**
P		2	2	**100**

* The data was extracted from HIV-1 sequence database (https://www.hiv.lanl.gov/components/sequence/HIV/search/search.html). "Hyper Vif" is defined as the sequence possessing F or Y at position 39 and H at position 48.
