# Supplementary material for: HIV-1 competition experiments in humanized mice show that APOBEC3H imposes selective pressure and promotes virus adaptation
Source: PLoS Pathog. 2017 May 5;13(5):e1006348. doi: 10.1371/journal.ppat.1006348 (PMC5435363; doi:10.1371/journal.ppat.1006348)
Supplement: S11 Table — A full list of the proportion of stable A3H individuals in each population and region from 1000 Genomes Project (http://www.internationalgenome.org). (PDF) [file ppat.1006348.s021.pdf]

Table S11. Proportion of stable A3H individuals in each population and region.

Population*	Region*	# Individual†				% Stable A3H‡
		Total	S/S	S/UI	UI/UI	
Americans of African Ancestry in SW USA	Africa	61	13	30	18	**70.5**
Utah Residents (CEPH) with Northern and Western Ancestry	Europe	85	1	15	69	**18.8**
Han Chinese in Bejing, China	Asia	97	2	13	82	**15.5**
Southern Han Chinese	Asia	100	1	13	86	**14.0**
Colombians from Medellin, Colombia	North America	60	3	10	47	**21.7**
Finnish in Finland	Europe	93	5	20	68	**26.9**
British in England and Scotland	Europe	89	0	25	64	**28.1**
Iberian Population in Spain	Europe	14	1	3	10	**28.6**
Japanese in Tokyo, Japan	Asia	89	0	16	73	**18.0**
Luhya in Webuye, Kenya	Africa	97	25	52	20	**79.4**
Mexican Ancestry from Los Angeles USA	North America	66	1	7	58	**12.1**
Puerto Ricans from Puerto Rico	North America	55	1	14	40	**27.3**
Toscani in Italia	Europe	98	2	17	79	**19.4**
Yoruba in Ibadan, Nigeria	Africa	88	35	37	16	**81.8**
	Total	1092	90	272	730	

* Population and region are defined in the website of 1000 Genomes Project.

† The data was extracted from 1000 Genomes Project (http://www.internationalgenome.org). S, stable (haplotypes II, V, and VII); UI, unstable (haplotypes III, IV, and VI) and intermediate (haplotype I).

‡ The individuals with S/S and S/UI are determined as "stable A3H".
