# Supplementary material for: HIV-1 competition experiments in humanized mice show that APOBEC3H imposes selective pressure and promotes virus adaptation
Source: PLoS Pathog. 2017 May 5;13(5):e1006348. doi: 10.1371/journal.ppat.1006348 (PMC5435363; doi:10.1371/journal.ppat.1006348)
Supplement: S12 Table — A full list of the proportions of hyper HIV-1 and stable A3H individuals in each region and country. (PDF) [file ppat.1006348.s022.pdf]

Table S12. Proportions of hyper HIV-1 and stable A3H individuals in each region and country.

Country/region*	# Individual*				**% Stable A3H†**	# Vif‡		**% Hyper Vif**
	Total	S/S	S/UI	UI/UI		# Total	# Hyper Vif	
Kenya	97	25	52	20	**79.4**	75	45	**60.0**
Nigeria	88	35	37	16	**81.8**	8	7	**87.5**
Spain	14	1	3	10	**28.6**	52	14	**26.9**
United Kingdom	89	0	25	64	**28.1**	11	5	**45.5**
Italy	98	2	17	79	**19.4**	3	0	**0**
United States	212	15	52	145	**31.6**	917	417	**45.5**
China	197	3	26	168	**14.7**	97	43	**44.3**
Japan	89	0	16	73	**18.0**	46	16	**34.8**
Africa					**78.0**			**68.8**
Europe					**23.5**			**37.3**
North America					**19.9**			**45.2**
Asia					**15.7**			**28.5**

* The data was extracted from 1000 Genomes Project (http://www.internationalgenome.org). S, stable (haplotypes II, V, and VII); UI, unstable (haplotypes III, IV, and VI) and intermediate (haplotype I). See also Table S11.

† The individuals with S/S and S/UI are determined as "stable A3H".

‡ The data was extracted from HIV-1 sequence database (https://www.hiv.lanl.gov/components/sequence/HIV/search/search.html). "Hyper Vif" is defined as the sequence possessing F or Y at position 39 and H at position 48.
